# Exploration of multiple Sortase A protein conformations in virtual screening

**DOI:** 10.1038/srep20413

**Published:** 2016-02-05

**Authors:** Chunxia Gao, Ivana Uzelac, Johan Gottfries, Leif A. Eriksson

**Affiliations:** 1 Department of Chemistry and Molecular Biology, University of Gothenburg, 405 30 Göteborg, Sweden

## Abstract

Methicillin resistant *Staphylococcus aureus* (MRSA) has become a major health concern which has brought about an urgent need for new therapeutic agents. As the *S. aureus* Sortase A (SrtA) enzyme contributes to the adherence of the bacteria to the host cells, inhibition thereof by small molecules could be employed as potential antivirulence agents, also towards resistant strains. Albeit several virtual docking SrtA campaigns have been reported, no strongly inhibitatory non-covalent binders have as yet emerged therefrom. In order to better understand the binding modes of small molecules, and the effect of different receptor structures employed in the screening, we herein report on an exploratory study employing 10 known binders and 500 decoys on 100 SrtA structures generated from regular or steered molecular dynamics simulations on four different SrtA crystal/NMR structures. The results suggest a correlation between the protein structural flexibility and the virtual screening performance, and confirm the noted immobilization of the β6/β7 loop upon substrate binding. The NMR structures reported appear to perform slightly better than the Xray-crystal structures, but the binding modes fluctuate tremendously, and it might be suspected that the catalytic site is not necessarily the preferred site of binding for some of the reported active compounds.

*Staphylococcus aureus* plays a leading role in hospital- and community-acquired infections which produces a wide spectrum of diseases ranging from minor skin infections, lower respiratory tract infections, surgical site infections, and nosocomial bacteremia, to pneumonia and cardiovascular infections^1^,^2^. The widespread occurrence of methicillin-resistant *S. aureus* (MRSA), which in addition to methicillin often is resistant to other commonly used antibiotics^3^, makes treatment difficult. This creates an urgent need for new therapeutic agents to treat MRSA infections, preferably ones that do not lead to rapid emergence of drug-resistant strains. A potential and attractive approach, which recently has gained much attention in treating these types of infections, is to inhibit surface proteins that function as virulence factors, with small molecules^4^.

*S. aureus* and many other Gram-positive pathogens use sortase A (SrtA) enzymes to anchor surface proteins to their cell walls^5^,^6^,^7^. This cysteine transpeptidase catalyzes the formation of an amide bond between a cell wall sorting signal (LPXTG motif) located at the C-terminal end of the surface protein, and a pentaglycine unit of the cell wall molecule lipid-II, thereby covalently attaching the surface protein to the cell wall^7^. Many surface proteins attached to the cell wall by SrtA play key roles in the infection process by promoting nutrient acquisition from the host, bacterial adhesion, and immune evasion^6^. Disrupting the display of these proteins by blocking the activity of SrtA using small molecule inhibitors could therefore effectively reduce bacterial virulence and thus promote bacterial clearance by the host. In addition, SrtA inhibitors may also be less likely to induce selective pressure that leads to drug resistance as compared to conventional antibiotics. This is supported by the fact that SrtA-lacking strains do not exhibit impaired growth outside of their human host in culture medium^8^, while at the same time altered adhesion properties has been observed^9^. To this end, a number of different strategies have been employed to search for sortase inhibitors. These include screening natural products^10^,^11^,^12^,^13^,^14^,^15^,^16^,^17^,^18^,^19^ and small compound libraries^20^,^21^,^22^,^23^, as well as synthesizing rationally designed peptidomimetics and small molecules^24^,^25^,^26^,^27^. To date, however, no SrtA inhibition based antibiotics have emerged on the market.

The structures of SrtA in its apo- and substrate-bound forms have been determined^28^,^29^,^30^. NMR and X-ray crystallography experiments have shown that the catalytic domain of SrtA (Residues 60–206) adopts a unique eight-stranded β-barrel fold with individual strands that are connected by two short helices and several loop regions (Fig. 1)^29^,^30^. Residues within the loop connecting the ß6 and ß7 strands (Residue 161–176) exhibit resonance line broadening in the NMR experiments, and were poorly resolved with high B-factors in the crystallography experiments. Both these findings indicate that this loop exhibits increased dynamics relative to the remainder of the protein. Motions of the ß6/ß7 loop are particularly interesting, given that many of its residues are positioned adjacent to the active site, notably residues 164–169. The active sites of all sortases contain a conserved catalytic triad that consists of residues H120, C184, and R197 (MRSA numbering), mutations to each of which have been shown to severely reduce the catalytic activity^31^.

In the holo-SrtA (substrate-bound SrtA) X-ray crystal structure, the LPXTG peptide substrate adopts an elongated form while the ß6/ß7 loop remains in an “open” conformation (Fig. 1D). R197 is observed to make contact with the LPXTG threonine residue; however, the side chain of the catalytic H120 is located more than 10 Å away from the peptide. In the holo-SrtA NMR structure, on the other hand, the LPXTG peptide analogue adopts an “L-shape” configuration in which there is a 90^o^ bend between the leucine and proline residues, and the ß6/ß7 loop not only contains a short 3_10_ helix spanning residues V166–L169, but is also far less mobile and in a “closed” configuration (Fig. 1B). To accommodate the covalently bound peptide in the active site, the ß7/ß8 loop adopts a more open conformation, suggesting an “induced-fit” mechanism for LPXTG peptide binding. In addition, each of the catalytic triad residues are observed in close proximity to residues within the LPXTG analog. Both the X-ray and NMR apo-SrtA structures adopt conformations that are similar compared to the holo-SrtA crystal structure, where the ß6/ß7 loop remains in an “open” conformation and the side chain of the catalytic H120 is located more than 10 Å , away from the LPXTG peptide. However, some fluctuations are observed in the ß6/ß7 and ß7/ß8 loops among these structures (Fig. 1A,C). Overall, the crystal and NMR structures of the apo- and holo-SrtA enzymes demonstrate large discrepancies in and around the active site, especially in the location of the conserved catalytic triad, and in the ß6/ß7 and ß7/ß8 loop conformations, which illustrate the large flexibility of the receptor structure.

Attempts have been made to employ reported receptor structures in virtual screening campaigns to identify new and efficient inhibitors against SrtA. In a virtual screening (VS) study which made use of the NMR structure of SrtA determined in its unbound state (apo-SrtA, PDB ID: 1IJA), the most active compound was subsequently found to have an IC50 value value of 58 μM^32^. In a more recent VS campaign employing a relaxed complex method for SrtA inhibitors, which made use of the NMR structure of SrtA bound to a substrate analog (holo-SrtA, PDB ID: 2KID), the most active compound was found to have an IC50 value of 47 μM^33^. Hence, neither of these VS efforts, conducted on receptor structures that differ considerably in the local geometry of the active site, resulted in finding inhibitors with medicinally relevant IC50 values. These unsatisfactory VS results can be due to the fact that the SrtA structure exhibits significant conformational heterogeneity and mobility as stated above. Simulation studies of the SrtA structure have furthermore shown that the active site of SrtA undergoes certain ligand-induced conformational changes^34^,^35^. Given that that neither SrtA nor other members of this protein family to date have been co-crystallized with any of the known non-covalent inhibitors, there is an urgent need for further exploration of the performance of flexible SrtA structures in finding truly good inhibitors. In other words, we need to better understand the receptor geometry, or ensemble of geometries, that is best employed in virtual screening campaigns in order to identify good inhibitors. In addition, the likely binding modes of small molecules at either the catalytic site or other areas of the protein, need be to carefully identified.

In the current work, we generated multiple molecular dynamics (MD) snapshots of apo- and holo-SrtA to represent a flexible ensemble of receptor coordinates for molecular docking. Based on previous experimental studies, which revealed a series of hits with good IC50 values, we constructed a library of 510 compounds, including 500 decoys and ten actives^11^,^13^,^14^,^15^,^18^,^20^,^21^. The ten active compounds have been concluded experimentally to be reversible (non-covalent) binders, even though some do contain rhodanine and other reactive functionalities. For example, the reversibility of the potentially covalent ligands 508-510 (Fig. 2) were determined using enzymatic assays and mass spectrometry. Ligand 508 is rapidly reversible as 84% of the enzyme activity is recovered immediately upon dilution; for ligands 509 and 510, the corresponding numbers are 50% and 58%, respectively. All active ligands selected herein have weak to moderate inhibitory effects (IC50-values 4 – 37 μM), and were chosen so as to be as structurally diverse as possible among the published SrtA inhibitors. The library of 510 compounds was screened against a total of 80 receptor models generated from the MD simulations, displaying considerable variability in the local structure. In addition, further conformational sampling was performed based on steered MD (SMD) simulations focusing on the flexibility of the β6/β7 loop, and docking performed on 20 snapshots along the SMD pathway. Through evaluation of the enrichment factors (EF)^36^, the Robust Initial Enhancement (RIE)^37^, the Boltzmann-Enhanced Discrimination of ROC metric (BEDROC)^38^, and the area under the Receiver Operating Characteristic curve (AUC)^39^, we systematically evaluate the relative VS performance of the various receptor models, also including a Boltzmann weighted average of the docked structures. The approach taken should hence be able to reliably identify the actives amongst the decoys, if binding towards the catalytic site is the only determining factor.

## Methods

### Structures and initial preparation

Four structures of SrtA (PDB ID: 1T2W, 1T2P, 2KID, 1IJA)^28^,^29^,^30^ were obtained from the protein data bank (www.rcsb.org), representing the crystal structure of C184A-mutated SrtA in complex with the LPETG (Leu-Pro-Glu-Thr-Gly) peptide; the crystal structure of SrtA in its apo state; the NMR structure of SrtA in complex with an LPXTG analog (X = Ala); and the NMR structure of SrtA in its apo state, respectively. Of these, the two NMR structures (2KID and 1IJA) were as mentioned above recently employed in VHTS campaigns identifying molecules that showed IC50 values in the 50 μM range^32^,^33^. As part of the receptor preparation, the mutated residue Ala184 in the 1T2W crystal structure was changed to Cys184. The termini of the LPXTG peptides were neutralized, and the calcium ion present in the 2KID structure retained. The four structures were then processed using the default Protein Preparation wizard in the Schrodinger programme suite before MD simulation. The protein preparation steps are described in detail below.

The charges of the LPXTG analog in the NMR holo SrtA structure were calculated using the restrained electrostatic potential (RESP) procedure^40^ at the HF/6-31G* level after minimizing the molecule at the AM1 semiempirical level^41^. GAFF force field33 parameters^42^ and RESP partial charges were assigned using the Antechamber module in the AMBER10 package^43^.

### MD simulations

The four structures prepared above were subjected to MD simulations, in order to obtain an ensemble of protein structures. Four conventional 200 ns MD simulations were performed on X-ray crystal apo- and holo-SrtA, and NMR apo- and holo-SrtA using the Amber 99 force field^44^,^45^ with the GROMACS software^46^. The structures were solvated in periodic boxes with a buffer distance of 10.0 Å. A number of Na^+^ and Cl^−^ ions were added to satisfy the electroneutrality condition and to give a salt concentration of 0.1 mol/liter, using the genion module in GROMACS. The obtained systems were energy minimized by steepest descent (200 steps) to remove close contacts. Position restrained simulations (2 ns duration, 1.0 fs time step, NPT ensemble, T = 298 K, P = 1 bar) were first performed, to enable the water molecules to reach more favorable positions. Particle-mesh Ewald (PME)^47^,^48^ summation was used for long-range electrostatics. A 12 Å cutoff was used for both Coulomb and Lennard-Jones interactions. The temperature and pressure was controlled through the Berendsen coupling algorithm^49^, with the time constants 0.1 for temperature and 1.0 ps for pressure coupling. All bond lengths were constrained using the LINCS algorithm^50^. During the production simulations (200 ns duration, 1.0 fs time step, NPT ensemble, T = 298 K, P = 1 bar), the temperature was controlled using the Nose-Hoover thermostat^51^ with a time constant 0.1 ps, and the pressure was controlled using the Parrinello-Rahman barostat^52^, with a time constant 1.0 ps. The remaining parameters were the same as in the position restrained simulations.

Steered MD (SMD) simulations were performed to further sample the conformational space around the flexible β6/β7 loop. In the SMD simulation, an external force (spring constant) of 10 kJ/mol/nm^2^ was added with pull rate 0.001 nm/ps, in order to move the β6/β7 loop from ‘closed’ to ‘open’ conformation. Using the distance between V166-L169 of the β6/β7 loop and C184 of the catalytic region of the active site as reference, a 17.5 ns SMD simulation was performed after a 2 ns position restrained equilibration MD, during which the β6/β7 loop was pulled away from the core structure thereby increasing the said distance from around 15 to 30 Å. Since the largest distance among the structures reported in the literature is around 22.5 Å (apo-SrtA crystal structure), the SMD has sampled a considerably larger set of conformations than those of the available reported structures. A total of 20 snapshots were extracted from the SMD, and ligand docking performed and analysed on these additional structures in the same manner as in the regular MD simulations.

### Decoy generation

The decoy generation methodology employed in the current study has been described previously^53^. Briefly, 10 actives were seeded among 2 million molecules from the ZINC database of commercially available compounds. Key feature fingerprints were calculated using the default type 2 substructure keys of CACTVS^54^, and fingerprint-based similarity analysis was performed with the programme Canvas. Compounds with Tanimoto Cofficient (Tc) less than 0.5 to any actives were selected. In the next step, QikProp (Schrodinger, LLC, New York, NY) was used to calculate 32 physical properties of all the actives as well as the selected ZINC compounds from the previous step and QikSim was applied to prioritize the ZINC compounds according to the properties of the actives. A weight of 4 was used to emphasize the druglike descriptors (molecular weight, number of hydrogen bond acceptors, number of hydrogen bond donors, number of rotatable bonds, and log P), and a weight of 1 was used for the number of important functional groups (amine, amide, amidine, and carboxylic acid). The rest of the descriptors were ignored (weight 0) during the similarity analysis procedure. After the calculation, 50 decoy compounds were selected for each active, leading to a total of 500 decoys that were physically similar but topologically dissimilar to the 10 actives. The 10 actives were selected based on their structures being as diversely distributed as possible among the known published SrtA inhibitors while at the same time being non-covalent inhibitors (Fig. 2). Selected properties of actives and the generated decoys are summarized in Table 1.

### Protein preparation and receptor grid generation

Twenty snapshots with evenly spaced intervals were extracted from each MD trajectory (including the SMD simulation) and prepared with the default protein Preparation wizard in Schrödinger. The protein preparation was carried out in two steps, preparation and refinement. After ensuring chemical correctness, hydrogen atoms were added, and side chains far from the binding cavity and not participating in salt bridges were neutralized. The hydrogen bonding network was optimized by reorienting hydroxyl groups, water molecules, and amide groups of Asn and Gln, and selecting appropriate states and orientations of the imidazole ring in His residues. Water molecules in the crystal structures were deleted, the termini were capped by adding ACE and NMA residues, and missing side chains and loops were added. The structures were then energy minimized using the OPLS-2005 force field^55^ and the Impact molecular mechanics engine, while heavy atoms were constrained. The thereby prepared protein structures were used for the subsequent step of grid generation.

**Figure 1:**
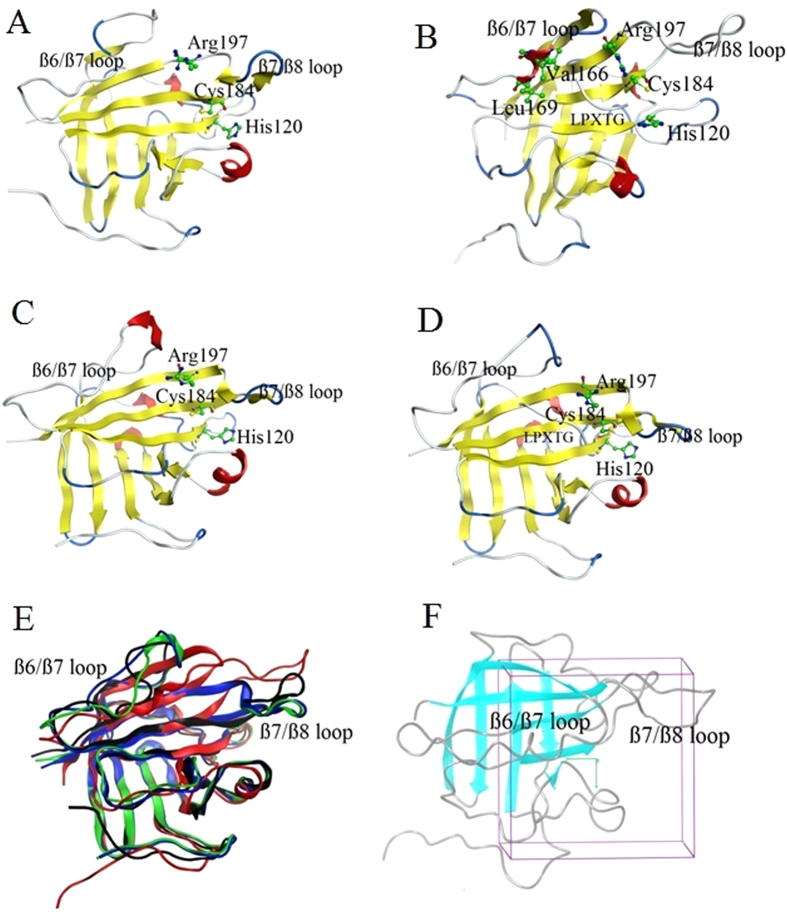
(**A**) Apo-SrtA NMR structure (PDB ID: 1IJA); (**B**) holo-SrtA NMR structure (PDB ID: 2KID); (**C**) apo-SrtA crystal structure (PDB ID: 1T2P) (**D**) holo-SrtA crystal structure; (**E**) superposition of all four SrtA structures, black ribbon: apo-SrtA NMR structure; red ribbon: holo-SrtA NMR structure; green ribbon: apo-SrtA crystal structure; blue ribbon: holo-SrtA crystal struture. (**F**) The binding site of SrtA is confined to the enclosing box used in the docking studies.

Grids were generated by the Receptor Grid Generation panel (Schrodinger, LLC, New York, NY) which defines the receptor structure by excluding any co-crystallized ligand present, determines the position and size of the active site to be represented by receptor grids, and sets up Glide constraints. Grids were defined by centering them on the ligand for the holo-SrtA structure using the default box size (box length 10 Å in the x-, y- and z-directions, respectively), and selecting site points generated by sitemap for the apo-SrtA structure (Fig. 1F). All docking was thus carried out in the region defined by the LPXTG substrate binding.

### Docking and scoring functions

Molecular docking experiments were carried out using Glide^56^, implemented in the Schrödinger package. The XP (extra precision) scoring functions were used, granting full flexibility to the ligands. A post-docking minimization was performed on the resulting complexes in order to reduce the initially collected 10000 poses per ligand to 5.

### Virtual screening performance analysis

Several metrics are available for evaluating the effectiveness of a docking run in discriminating actual binders from decoys. For evaluating the performance of different combinations of protein conformers, we considered EF^36^, RIE^37^, BEDROC^38^ and AUC^39^.

EF is a widely used metric to evaluate the efficiency of VS^36^. The value of EF^x%^ indicates how much more often an active compound is ranked in the top x% of a VS result compared to a random selection, i.e., how many times the database is enriched. Specifically, EF is calculated according to Eq. 1:


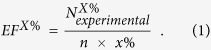



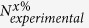
is the number of experimentally verified actives in the top x% of the database and 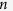
is the total number of actives in the database. In this study, EF1% was calculated from the top 1% of the docking results.

We also report the BEDROC and RIE values to explore the problem of “high scoring”^58^. By changing a tuning parameter, *α*, users can control the level of top scoring actives to test whether a certain ranking method is useful in the context of VS. BEDROC is bound by the interval [0, 1] and can be interpreted as the probability that an active is ranked before a randomly selected compound exponentially distributed with parameter *α*, given that 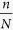
*α *≪ 1 (*n* = number of actives; *N* = total number of compounds). RIE uses an exponential weighting scheme that gives heavier weight to “top scoring” actives as defined in Eq. 2:


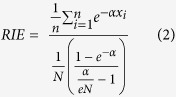


where *x*_*i*_ = 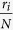 is the relative rank of the *i*th active and α is a tuning parameter. BEDROC is derived from RIE and has a linear relationship with RIE as shown in Eq. 3:


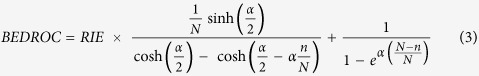


Although RIE and BEDROC produce different values, their distributions scale very similarly.

To investigate the docking performance in a threshold independent manner, the AUC value was calculated from the ROC curve. The ROC curve allows a straightforward visualization of the performance of VS in ranking the actives higher over decoys^39^. In our study, we have a list of experimentally verified actives (positives), and decoys (negatives). These positives and negatives are further categorized into true or false according to their rank above or below a certain threshold of the VS result, i.e., the actives ranked above a chosen threshold becomes true positive (TP). To generate the ROC curve, the true positive ratio (TPR) and false positive ratio (FPR) are calculated as:


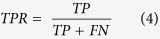



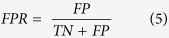


In the ROC curve, the TPR is plotted as a function of the FPR. The AUC is then calculated to assess the quantitative performance of different receptor models. An AUC of 0.5 corresponds to a random selection of the ligands by the receptor^57^.

## Results and Discussion

### MD simulations

Due to the large discrepancies between the reported SrtA structures, MD simulations were performed on four initial structures representing apo- and holo-SrtA NMR and crystal structures in order to fully explore the flexibility of the receptor conformations for the docking study. To assess how the conformations varied during each simulation, we calculated the Cα root mean square deviations (RMSDs) relative to the initial structure, Cα root mean square fluctuations (RMSFs), and the radius of gyration of the proteins.

The RMSD of the apo-SrtA NMR structure in the simulation stabilized at ~3.5 Å after 10ns; the holo-SrtA NMR structure stabilized at ~2.5 Å in the first 25 ns, then went up to ~3.5 Å after 45 ns; the apo-SrtA crystal structure described almost the same fluctuation as the holo-SrtA NMR structure, except for a reduction down to ~2 Å after 175 ns; and the holo-SrtA crystal structure was stabilized after 5 ns at ~1.5 Å (Fig. 3A). Hence, the holo-SrtA crystal structure appears to be the one in the best thermal equilibrium, although at the same time its β6/β7 loop is displaced the most from the active site.

It can be seen from the RMSF of the Cα atoms (Fig. 3B) that residues within the loop regions are quite dynamic, especially the ß6/ß7 loop in both the apo-SrtA NMR and crystal structures, i.e. without peptide binding; however, the RMSF of the ß6/ß7 loop in the holo-SrtA structures are relatively low, and hence, the dynamics of the ß6/ß7 loop appears to be affected by the peptide binding. This confirms a previous study where it was reported that the peptide binding immobilizes the loop^28^.

The radius of gyration of a protein is calculated as the root mean square distance between its center of gravity and its ends, and is indicative of the level of compactness of the structure. It can be seen (Fig. 3C) that the radius of gyration is quite stable for each structure during the simulation time, with the holo-SrtA NMR structure being less compact compared to other structures, due to the more extended form of the ß7/ß8 loop. Overall, in terms of Cα RMSDs, RMSFs, and the protein radius of gyration calculations, the conformation of each structure was stable during in the simulation, with the loop regions as expected displaying the largest fluctuations.

To analyse the active site of the receptor structures, the all-atom RMSDs of the active site residues 90–112, 120–130, 161–176 and 183–196; the distance between the center of mass of residues V166–L169 and the catalytic residue C184; and the number of hydrogen bonds between R197 and the LPXTG peptide, respectively, were calculated.

The all atom RMSDs of the active site residues, including side chains, were calculated to investigate their flexibility. The active site residues displayed large flexibility in the apo-SrtA NMR structure during the simulation time, where the RMSD stayed at ~3Å within the first 60 ns, then rose to 4 Å within 60–100 ns, and after 100 ns to 5 Å for the remaining of the simulation. In the holo-SrtA NMR structure simulation, the RMSD of the active site residues stays mostly at ~3.5 Å, and arises to 4 Å after 175ns, whereas in the apo-SrtA crystal structure simulation, the RMSD of the active site residues rises to 3 Å within the first 25 ns, and then rises to 5 Å till 100 ns, stays at ~5.5 Å from 100 ns to 175 ns, and drops down to 4 Å. In the holo-SrtA crystal structure simulations, finally, the RMSD of the active site residues remains more stable around 2.5 Å in the whole simulation (Fig. 4A). The RMSDs of the active site residues demonstrate that the binding sites of the apo structures of SrtA are more flexible than the holo structures, which again confirms that the LPXTG peptide binding stabilizes the active site.

The distance between the center of mass of residues V166– L169 and C184 was calculated to see how the ß6/ß7 loop fluctuates relative to the catalytic residues (Fig. 4B). The distance in the apo-SrtA crystal structure quickly rises to ~20 Å, whereas in the holo-SrtA crystal structure the distance remains at 17.5 Å. In the apo-SrtA NMR structure the distance drops from 17.5 Å to 12.5 Å, which is almost the same trend as in the holo-SrtA NMR structure. The distance changes indicate that, in the holo-SrtA structures, the loop keeps the same distance to C184 in the MD simulation as in the initial structure. The holo-SrtA NMR structure thus retains a more compact active site structure, whereas the holo-SrtA crystal structure has a more ‘open’ active site structure. In the apo–SrtA structures, on the other hand, the distance fluctuates more due to the lack of substrate interaction with the loop region.

In the initial holo-SrtA (substrate-bound SrtA) X-ray crystal structure, the LPETG peptide substrate adopts an elongated form while the ß6/ß7 loop remains in an “open” conformation (Fig. 1D). R197 is observed to make contact with the LPETG threonine residue. In the holo-SrtA NMR structure, on the other hand, the LPATG peptide analog adopts an “L-shape” configuration in which there is a 90^o^ bend between the leucine and proline residues, and R197 makes contact with the LPATG alanine residue. During the MD simulations, those residues which are hydrogen bonding to the proteins keep still, whereas all other residues fluctuate significantly. The number of H-bonds in both the holo-SrtA structures fluctuates between 1 and 2 and sometimes even 3 during the simulation time, which indicates that the interaction between R197 and LPXTG is retained in both structures (Fig. 4C,D). The LPETG peptide substrate remains in its elongated form in the holo-SrtA X-ray crystal structure, whereas in the holo-SrtA NMR structure the “L-shape” configuration of the LPATG analogue disappears during the MD simulation.

### Enrichment calculations

Table 2, 3, 4, 5 show the EF^1%^, BEDROC (α = 160.9), BEDROC (α = 20), AUC, and RIE after docking the 510 molecules to the 80 receptor models extracted from the MD simulations of the apo- and holo-SrtA NMR and crystal structures, using the Glide-XP scoring function.

The enrichment factor EF^1%^ metric, which reflects the database enrichment performance in the top 1% of a library, becomes particularly relevant in assessing the predictive power of VS. As can be seen from Table 2, 15 out of 20 snapshots of the apo-SrtA NMR structure simulations have EF^1%^ = 10, 1 out of 20 have EF^1%^ = 20, and the remaining have EF^1%^ = 0, giving an average EF^1%^ of 8.5. Snapshots generated from the holo-SrtA NMR structure, the apo-SrtA crystal structure, and the holo-SrtA crystal structure MD simulations gave average EF^1%^ of 9, 2 and 5.5 respectively. Overall, the EF^1%^ ranges from 0 to 20 for the different SrtA conformations, indicating that the VS results are not particularly sensitive to the active site conformational changes; however, the EF^1%^ results also suggest that the receptor models generated from the apo- and holo-SrtA crystal structures have very poor enrichment performance. This is most likely due to the large and bulky space available for fitting the decoys into the active site, as discussed more below. In general, the decoys that ranked higher than the actives were of high molecular weight and had higher propensity to form hydrogen bonds to R197. In terms of BEDROC (α = 160.9) enrichments, the snapshots from the apo- and holo-SrtA NMR and crystal structures return averages of 0.155, 0.172, 0.049, and 0.1, respectively, whereas the BEDROC (α = 20) enrichments yield averages of 0.162, 0.179, 0.116 and 0.162. For the RIE enrichments, the corresponding averages are 2.68, 2.97, 1.92, and 2.68, respectively. In these scoring metrics, earlier parts of the hit list count more heavily in the enrichment as stated above. The exact reason for differences in enrichment results between neighbouring snapshots generated from the same initial structures are not entirely clear, since the structural changes are often small. Another observation from the tables is that the snapshots at 0 ns which represent the initial NMR or crystal structures do not generate the best results. This indicates that the *in vivo* SrtA ligand binding conformations are significantly different compared to the retrieved NMR/crystal based structure models leading to the corollary that MD simulations can be important in generating better structures to use in VS campaigns; or, alternatively, that the active site is in fact not the key binding area of (some of) the ligands.

When comparing the enrichment results to the structural analyses of section 3.1, there is a clear correlation between the average ß6/β7 loop distance to the catalytic residue C184 and the average EF^1%^ and the BEDROC (α = 160.9) values, where these two enrichment metrics counts more heavily on the high scoring binders in the VS. The distance measure demonstrates that the loop is in either “open” or “closed” conformation of the active site. The MD simulations generate a much more “open” conformation for both apo- and holo-SrtA crystal structures, but a more “closed” conformation for the apo- and holo-SrtA NMR structures with 2KID and 1IJA being the initial structures employed. Overall, the more “closed” conformation returns better EF^1%^ and BEDROC (α = 160.9) results (Fig. 5A). However, we found no correlation between the average ß6/β7 loop distance to the catalytic residue C184 and the average RIE or BEDROC (α = 20), or between the flexibility of the active site residues and any of the enrichment metrics (Fig. 5B).

Finally, a Boltzmann distribution function was applied to all the ensemble docking scores in order to obtain a weighted average score per ligand. Based on the Boltzmann weighted average docking score we calculated the average enrichment factors, BEDROC, AUC and RIE data; however, the results thus obtained do not provide any further improvement compared to using the ‘raw’ data for each snapshot (Suppplementary Table S6).

### Area under receiver operating characteristic (ROC) curves

The performance of each receptor conformation according to the AUC can be divided as excellent (above 0.9), moderate (0.9 to 0.6), and poor (less than 0.6). Compared to EF^1%^, BEDROC and RIE, which measure for high scoring performance, AUC sees to the overall performance in identifying the known binders. All the 80 receptor conformations generated from the MD simulations were indicated to perform poor or moderately well as per the AUC definition. The best AUC for the apo-SrtA NMR structure is 0.80, whereas 13 out of 20 snapshot conformations performed poor and 7 out of 20 performed moderately well, with an average AUC of 0.57; the best AUC for the holo-SrtA NMR structure is 0.78, 5 out of 20 performed poor and 15 out of 20 performed moderately well, with an average AUC of 0.64; the best AUC for the apo-SrtA crystal structure is 0.78, 13 out of 20 performed poor, 7 out of 20 performed moderately well, with an average AUC of 0.57; and the best AUC for the holo-SrtA crystal structure is 0.78, with an average AUC of 0.63 (ROC plots for all the snapshots are given as Supplementary Figure S1).

This unsatisfactory AUC performance of the receptor conformation was mainly due to the fact that some receptor conformations could bind certain ligands remarkably well but could not identify those which required a different binding site arrangement. This result further supports the argument that the SrtA structure undergoes ligand-induced conformational changes, possibly upon initial ligand binding, which indicates that the SrtA proteins comprise conformational variety that yet remains to be unfolded.

### Ligand docking poses and ranking

The docked active ligands in the initial receptor structures were examined to characterize important interactions in the SrtA binding site and the differences in docking poses. For Ligand_501, the orientation of the molecule differs between the four receptor structures used, but the molecule interacts in all cases with the three key residues His120, Cys184, Arg197. For Ligand_502, the binding pose in the apo-SrtA crystal structure is distinct and interacts mainly with Helix ß6/7, and for Ligand_503, the molecule is located in the center of the binding pocket in the holo-SrtA crystal structure instead of interacting with the key residues. Ligand_504 interacts via its carboxylic group with Arg197 in the apo- and holo-SrtA NMR structure and the apo-SrtA crystal structure, but not with holo-SrtA crystal structure. For Ligand_505, the binding to the holo-SrtA crystal structure is again different from the rest as it interacts more with Helix ß6/7. Ligand_506 has attains a similar docking pose in all receptor structures, and interacts with the key residues, Helix ß6/7 and Helix ß7/8, whereas for Ligand_507, the binding poses differ between all the receptors. The docking pose of Ligand_508 in the holo-SrtA NMR crystal structure is different compared to the results for the other three receptors, and the molecule only partly interacts with the three key residues. For Ligand_509, the interactions of the molecule display significant differences between the receptors, due to its small size enabling the molecule to fit readily at several places in the pockets. Ligand_510, finally, attains different orientations but the general interactions are similar in all four systems (Supplementary Figure S2). In general, the ten actives are mostly located adjacent to the key residues in the different receptors, although some do locate closer to the helixes and/or the center of the binding pocket.

**Figure 2:**
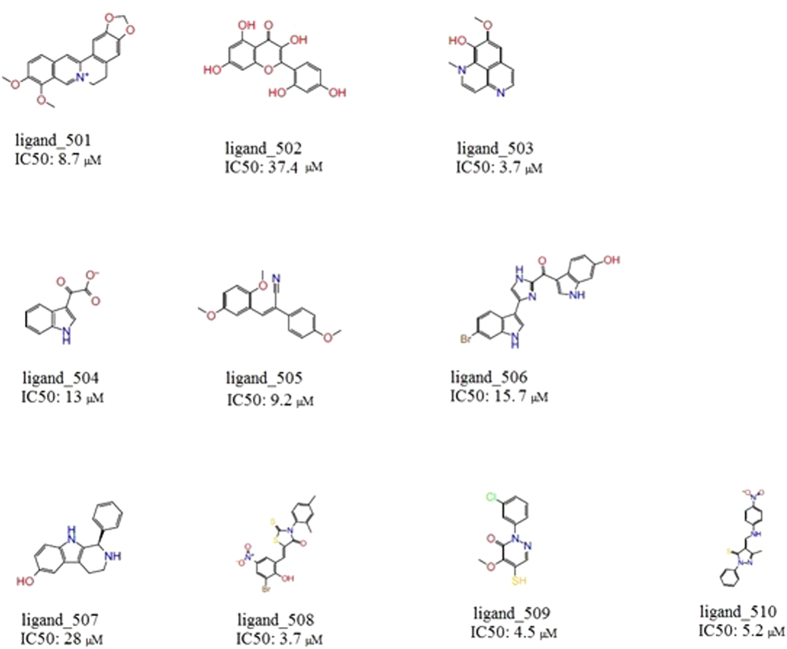
Active inhibitors with different IC50 values and diverse structures used as ‘known binders’ in this work.

**Figure 3:**
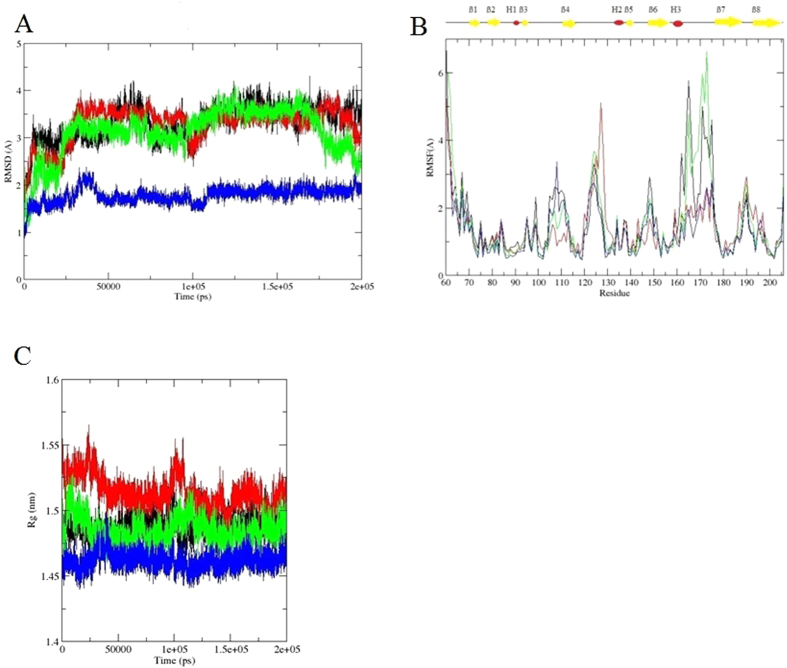
Data for the different SrtA structures over the 200 ns MD simulations. Line color represents: black: apo-SrtA NMR structure; red: holo-SrtA NMR structure; green: apo-SrtA crystal structure; blue: holo-SrtA crystal struture. (**A**) Cα RMSD; (**B**) Cα RMSFs; location of α-helices and β-strands indicated above the graph; (**C**) Radius of gyration. See text for further details.

**Figure 4:**
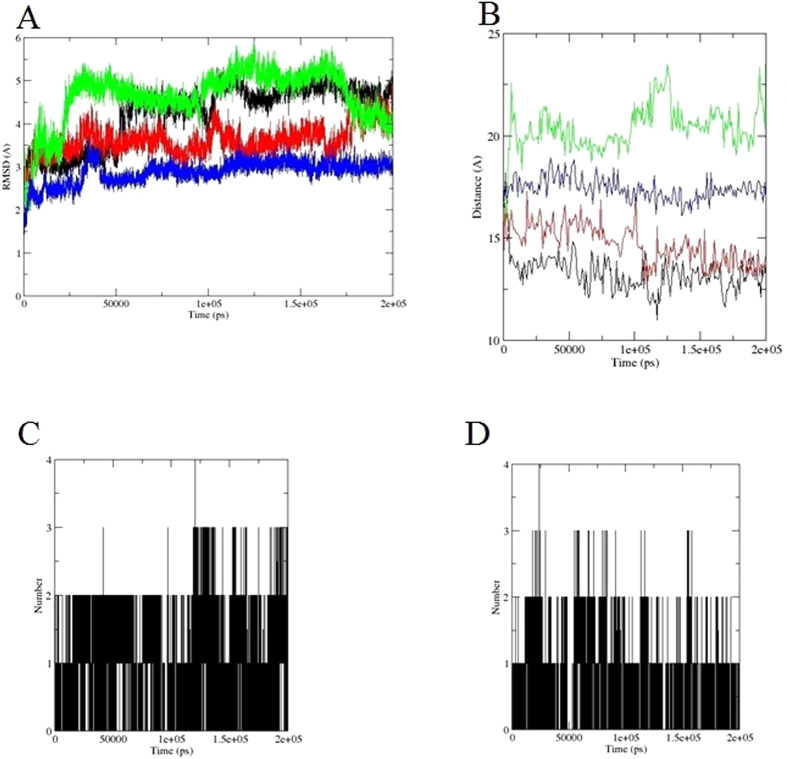
Active site parameters during the 200 ns MD simulations. Line colors as in Figure 3. (**A**) All atom RMSDs of the SrtA active site residues; (**B**) Distance between the center of mass of residue V166-L169 (part of β6/β7 loop) and C184; (**C**) Number of hydrogen bonds between R197 and LPXTG analog in holo-SrtA NMR structure; (**D**) As (**C**) but for LPXTG substrate in holo-SrtA crystal structure.

**Figure 5:**
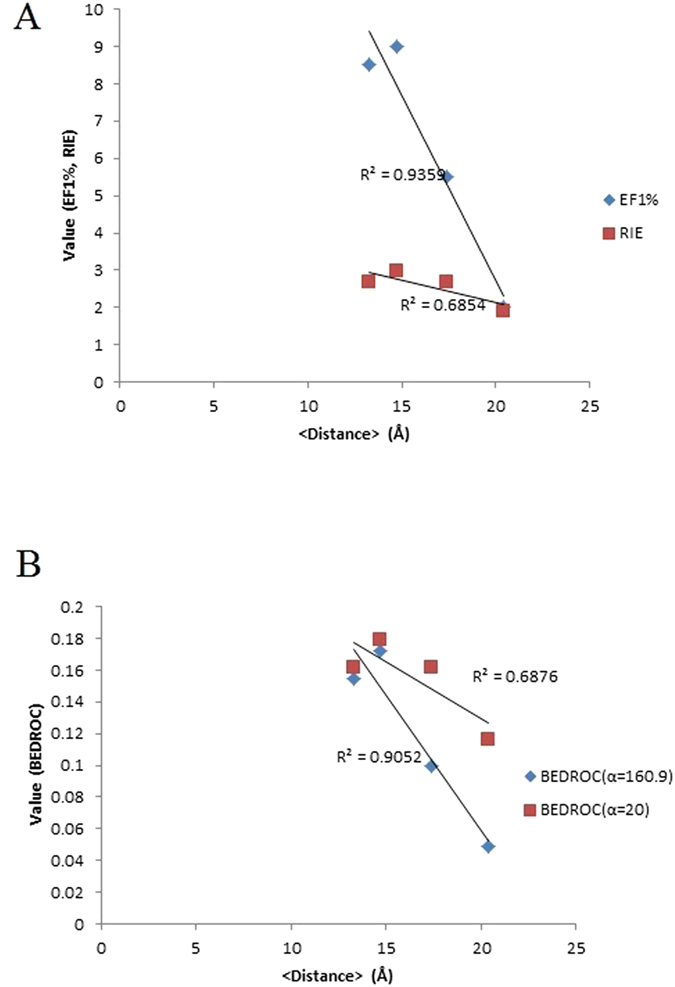
(**A**) Correlation between average distance of V166-L169 to C184 with EF^1%^ and RIE; (**B**) Correlation between average distance of V166-L169 to C184 with BEDROC (α = 160.9) and BEDROC (α = 20). See Supplementary Table S1 for data values.

As can be expected, the VS results confirm that true binders were ranked at the first positions when a suitable conformation was used. In this study, ligand_502 (*cf.* Fig. 2) ranked 14 times, ligand_506 ranked 5 times, and ligand_507 1 time in the top 1% of the VS results of holo-SrtA NMR structures; ligand_502 ranked 17 times, ligand_504 ranked 1 time in the top 1% of the VS results of apo-SrtA NMR structures; ligand_502 ranked 3 times time in the top 1% of the VS results of apo-SrtA crystal structures; and ligand_502 ranked 7 times, ligand_506 ranked 2 times in the top 1% of the holo-SrtA VS results of crystal structures (Table S2–S5). Overall, ligand_502, which is the well-known active compound morine, ranked the best amongst the known binders in the MD simulation snapshots generated from different starting structures, with some snapshots also seen to provide good geometries for interaction with ligand_504, ligand_506 and ligand_507. However, the overall poor performance in the ranking, with on average 1, and at the most 2 of the reported good binders ending up among the top 10 ranked compounds, irrespective of initial structure or snapshot, indicates that the catalytically active site may not necessarily be the preferred site of interaction for (several of) these compounds. Alternatively, the geometric features of the active site capable of discriminating that these compounds actually do bind better than the decoys, have as yet to be identified. From the above results, SrtA active site virtual screening campaigns using either of the reported Xray/NMR structures more or less “as is”, with the aim to generate novel inhibitors, are essentially bound to fail. A further observation that can be made is that the ranking of the ligands does not correlate with the trends in reported IC50 values, and that large variations in the set are noted, from one snapshot to another. For example, ligand 502 (morine) is ranked number 2 in the 20 ns snapshot starting from the apo-form of the SrtA crystal structure, but as number 324 (out of 510) in the snapshot at 25 ns.

### Results from SMD simulations

Although MD simulations can provide snapshots that are different from the crystal structures, they did in this particular case not generate a wide enough diversity of conformations to provide good binding to all the known ligands. In most cases, the extent of MD sampling will determine the diversity of conformations, relying on how well the sampling has explored phase space. Enhanced sampling methods, such as accelerated molecular dynamics and replica exchange simulations, that allow for more rapid exploration of conformational space might be useful in this respect. In order to cover a larger conformational region, steered MD simulations were performed in the current study, in which the β6/β7 loop was gradually shifted from ‘closed’ to ‘open’ conformation by pulling the V166-L169 segment of the loop away from the catalytically active C184. From the SMD trajectory, 20 snapshots were used in docking simulations and analyses. In Supplementary Figure S3 the distance variation between the β6/β7 loop and C184 during the 17.5 ns SMD simulation is displayed, and in Supplementary Table S7, the resulting EF^1%^, BEDROC, AUC and RIE data from the 20 docking runs are collected. The best AUC for the SMD snapshots is 0.78, whereas 2 out of 20 snapshot conformations performed poor and 18 out of 20 performed moderately well, with an average AUC of 0.67; 1 out of 20 snapshots of the SMD structure simulations have EF^1%^ = 0, 5 out of 20 have EF^1%^ = 20, and the remaining have EF^1%^ = 10, giving an average EF^1%^ of 12; the average BEDROC (a = 160.9), BEDROC(a = 20.0) and RIE values are 0.246, 0.21 and 3.73 correspondingly. Compared to the values from the ‘normal’ MD snapshots described above, the SMD snapshots perform somewhat better but provide no further improvement in recognizing the true actives from the decoys. The snapshots from 15.5 ns and onwards have a distance between the V166-L169 and C184 larger than 22.5 Å (See Figure S3), and hence represent ‘extremely open’ geometries not present in the reported structures. However, no marked improvements in data were seen in those snapshots despite sampling conformations not accessible from the normal MD trajectories.

## Conclusions

In this work, we have presented an exploratory analysis of VS performance for apo- and holo-SrtA based on molecular dynamics snapshots with diverse molecular flexibility and binding site properties. The MD simulation studies demonstrate that the SrtA structure is dynamic, especially in the loop regions, and that the LPXTG substrate on binding can induce distinct loop conformation differences in the binding site. When considering a compound binding to the active site of SrtA, it thus appears important to take into account the motion of the active site residues in the loop, particularly those located in the β6/β7 and β7/β8 loops. The analysis of early enrichment metrics and the AUC for different receptor models showed that the best EF^1%^ is 20 and the best AUC is 0.8, demonstrating that the receptor models at best performed moderately well in distinguishing the actives from the decoys. This might imply that more thorough structural information, *e.g.*, regarding possible ligand induced fit and protein flexibility, is needed to unlock the SrtA binding modes to its inhibitors. Such knowledge would enhance the ligand-based screening accuracy and potentially yield a more positive outcome than structure-based screening in this sense. Comparison of the average distance between the β6/β7 loop and C184, and the EF^1%^ and BEDROC (α = 160.9) metrics revealed a correlation between the more “closed” conformation and better scoring results. No correlation was found between the RMSDs of SrtA active site residues, BEDROC (α = 20), RIE and AUC. On the other hand, the results in this work suggest that MD conformations can improve VS results compared to the use of X-ray or NMR structures only. A general conclusion demonstrated by this study is that no single property could be used to separate a best receptor structure for the whole set of actives studied here. Using a Boltzmann weighted average score over the different snapshots did not provide any improvement, nor did the use of snapshots from a SMD simulation tailored at changing the system from ‘closed’ to very ‘open’ with respect to the β6/β7 loop. Although it is possible that even more structurally diverse conformations would increase the likelihood of improving the correlation with experiments, and to help discovering truly novel scaffolds, conformational space around the active site has in the current work been covered rather extensively. Alternatively, the actual binding mode may for several of these compounds in fact be located away from the active site, which would also explain the ‘randomness’ of the data obtained in the current work. In that sense, the current work also challenges the accuracy of the reported SrtA binding compounds. Finally, in a full VS campaign, where high scoring actives is often crucial, EF^1%^ and BEDROC (α = 160.9) seem to be the most appropriate metrics to evaluate performance towards SrtA. It can also not be ruled out that due to the large and flexible active site region, peptide-mimetic based inhibitors might be more optimal binders in the SrtA active site, than small organic molecules.

## Additional Information

**How to cite this article**: Gao, C. *et al.* Exploration of multiple Sortase A protein conformations in virtual screening. *Sci. Rep.* **6**, 20413; doi: 10.1038/srep20413 (2016).

## Supplementary Material

Supplementary Information

## Figures and Tables

**Table 1:** Average properties of actives and decoys.

	**Compounds**	**Molecular weight**	**Rotable bonds**	**H-bond acceptors**	**H-bond donors**
Actives	10	310.84 ± 66.22	2.5 ± 1.2	3.2 ± 1.0	1.7 ± 1.6
Decoys	500	303.30 ± 59.21	3.6 ± 1.3	2.5 ± 0.9	1.0 ± 0.9

**Table 2:** EF^1%^, BEDROC (α = 160.9), BEDROC (α = 20), AUC, RIE values for snapshots of the apo-SrtA NMR structure MD simulations.

MD snapshot (ns)	EF^1%^	BEDROC (a = 160.9)	BEDROC (a = 20.0)	AUC	RIE
0	10	0.206	0.181	0.58	2.99
5	0	0.012	0.099	0.51	1.64
10	20	0.392	0.255	0.46	4.22
20	0	0.031	0.097	0.39	1.61
40	10	0.08	0.121	0.5	2
60	10	0.237	0.231	0.52	3.83
70	10	0.21	0.25	0.8	4.13
80	10	0.283	0.16	0.6	2.64
90	10	0.151	0.192	0.77	3.18
100	10	0.11	0.118	0.62	1.95
110	10	0.162	0.192	0.6	3.18
120	10	0.209	0.213	0.54	3.53
130	10	0.08	0.11	0.56	1.81
140	0	0.031	0.106	0.56	1.76
150	10	0.206	0.145	0.52	2.4
160	10	0.081	0.177	0.62	2.93
170	10	0.132	0.245	0.7	4.05
180	10	0.283	0.196	0.49	3.23
190	10	0.206	0.134	0.55	2.22
200	0	0	0.015	0.49	0.25
Average	8.5	0.155	0.162	0.57	2.68

**Table 3:** EF^1%^, BEDROC (α = 160.9), BEDROC (α = 20), AUC, RIE values for snapshots of the holo-SrtA NMR structure MD simulations.

MD snapshot (ns)	EF^1%^	BEDROC (a = 160.9)	BEDROC (a = 20.0)	AUC	RIE
0	10	0.15	0.116	0.44	1.92
5	10	0.206	0.163	0.6	2.69
10	10	0.325	0.242	0.76	4.01
20	10	0.283	0.206	0.6	3.41
30	10	0.11	0.158	0.63	2.61
40	10	0.15	0.132	0.62	2.18
60	20	0.286	0.246	0.68	4.08
70	10	0.11	0.196	0.69	3.24
90	0	0.001	0.114	0.64	1.88
100	0	0.209	0.256	0.68	4.24
110	10	0.283	0.18	0.65	2.99
120	10	0.11	0.167	0.58	2.77
130	10	0.11	0.141	0.6	2.33
140	10	0.15	0.13	0.58	2.21
150	20	0.363	0.236	0.76	3.91
160	10	0.249	0.222	0.61	3.67
170	10	0.111	0.193	0.66	3.19
180	0	0.006	0.124	0.57	2.05
190	0	0.002	0.113	0.67	1.87
200	10	0.229	0.249	0.69	4.12
Average	9	0.172	0.179	0.64	2.97

**Table 4:** EF^1%^, BEDROC (α = 160.9), BEDROC (α = 20), AUC, RIE values for snapshots of the apo-SrtA crystal structure MD simulations.

MD snapshot (ns)	EF^1%^	BEDROC (a = 160.9)	BEDROC (a = 20.0)	AUC	RIE
0	10	0.152	0.181	0.63	2.99
5	10	0.291	0.289	0.75	4.77
10	0	0.043	0.1	0.58	1.65
20	10	0.209	0.231	0.78	3.82
25	0	0	0.004	0.41	0.07
30	0	0	0.003	0.42	0.05
40	0	0	0.064	0.52	1.06
70	0	0.009	0.1	0.64	1.66
90	0	0	0.06	0.5	0.99
100	0	0.004	0.122	0.63	2.01
110	0	0.01	0.147	0.48	2.42
120	0	0	0.059	0.54	0.97
130	0	0	0.046	0.49	0.76
140	0	0.009	0.091	0.42	1.51
150	10	0.206	0.181	0.69	3
160	0	0.032	0.284	0.73	4.7
170	0	0	0.103	0.58	1.7
180	0	0.024	0.186	0.54	3.07
190	0	0	0.005	0.54	0.09
200	0	0	0.062	0.53	1.02
Average	2	0.049	0.116	0.57	1.92

**Table 5:** EF^1%^, BEDROC (α = 160.9), BEDROC (α = 20), AUC, RIE value for snapshots of the holo-SrtA crystal structure MD simulations.

MD snapshot (ns)	EF^1%^	BEDROC (a = 160.9)	BEDROC (a = 20.0)	AUC	RIE
0	0	0.015	0.166	0.61	2.74
5	0	0.031	0.099	0.61	1.63
10	0	0.059	0.173	0.6	2.86
30	0	0	0.048	0.42	0.79
40	10	0.11	0.195	0.66	3.23
50	10	0.206	0.142	0.74	2.35
60	10	0.119	0.242	0.65	4.01
70	10	0.11	0.17	0.57	2.82
90	0	0.033	0.156	0.63	2.58
100	10	0.11	0.121	0.62	1.99
110	0	0.047	0.198	0.65	3.28
120	10	0.159	0.225	0.78	3.72
130	0	0.089	0.196	0.66	3.25
140	20	0.489	0.236	0.64	3.91
150	10	0.141	0.202	0.7	3.35
160	0	0.01	0.162	0.7	2.69
170	10	0.08	0.163	0.61	2.69
180	10	0.283	0.174	0.6	2.88
190	0	0.001	0.106	0.62	1.75
200	0	0.002	0.071	0.57	1.18
Average	5.5	0.105	0.162	0.63	2.69
