# Supplementary material for: Exploration of multiple Sortase A protein conformations in virtual screening
Source: Sci Rep. 2016 Feb 5;6:20413. doi: 10.1038/srep20413 (PMC4742773; doi:10.1038/srep20413)
Supplement: Supplementary Information [file srep20413-s1.pdf]

Exploration of multiple Sortase A protein conformations in virtual screening

Chunxia Gao, Ivana Uzelac, Johan Gottfries, Leif A Eriksson[*]

Department of Chemistry and Molecular Biology, University of Gothenburg,

405 30 Göteborg, Sweden

SUPPLEMENTARY DATA:

Figures S1-S3

Tables S1-S7

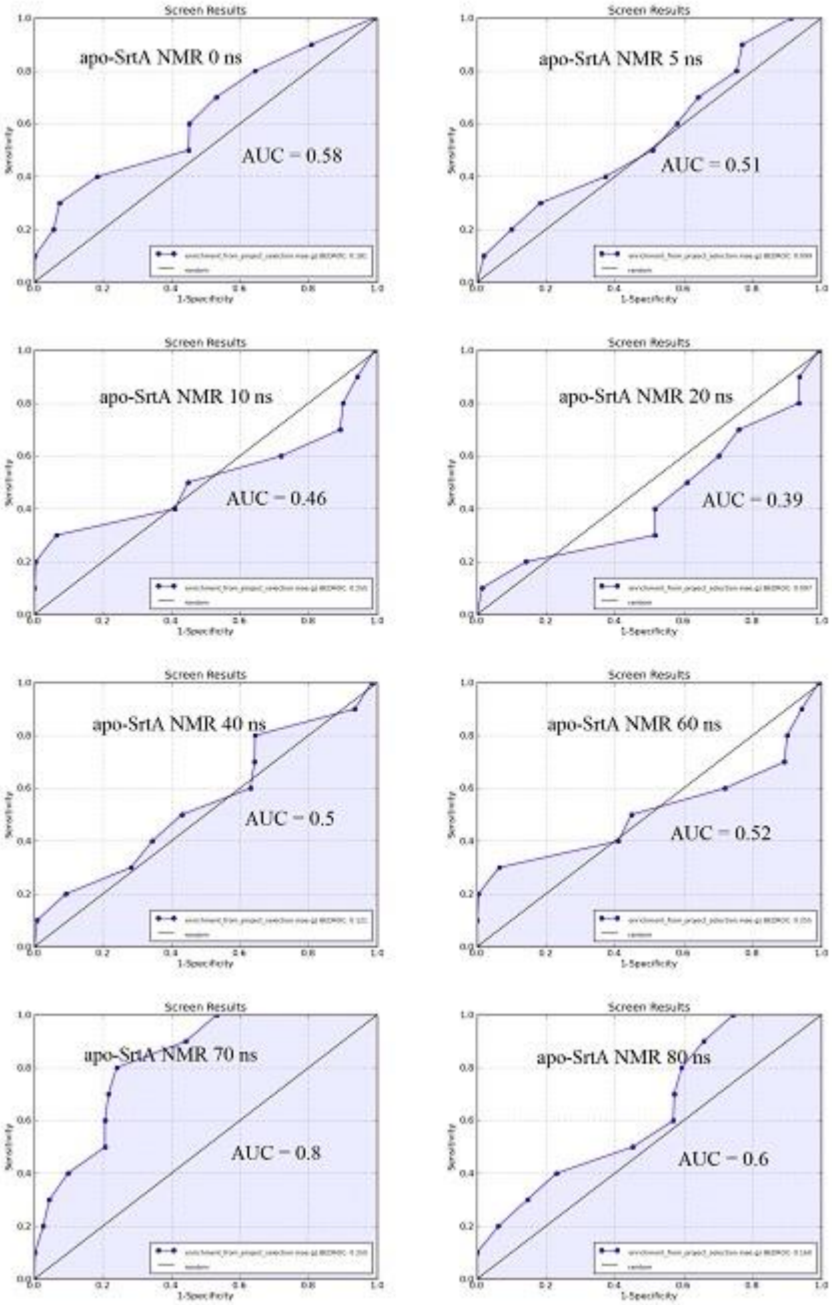

Screen Results
apo-SrtA NMR 0 ns
AUC = 0.58
Screen Results
apo-SrtA NMR 5 ns
AUC = 0.51
Screen Results
apo-SrtA NMR 10 ns
AUC = 0.46
Screen Results
apo-SrtA NMR 20 ns
AUC = 0.39
Screen Results
apo-SrtA NMR 40 ns
AUC = 0.5
Screen Results
apo-SrtA NMR 60 ns
AUC = 0.52
Screen Results
apo-SrtA NMR 70 ns
AUC = 0.8
Screen Results
apo-SrtA NMR 80 ns
AUC = 0.6
Sensitivity
1-Specificity

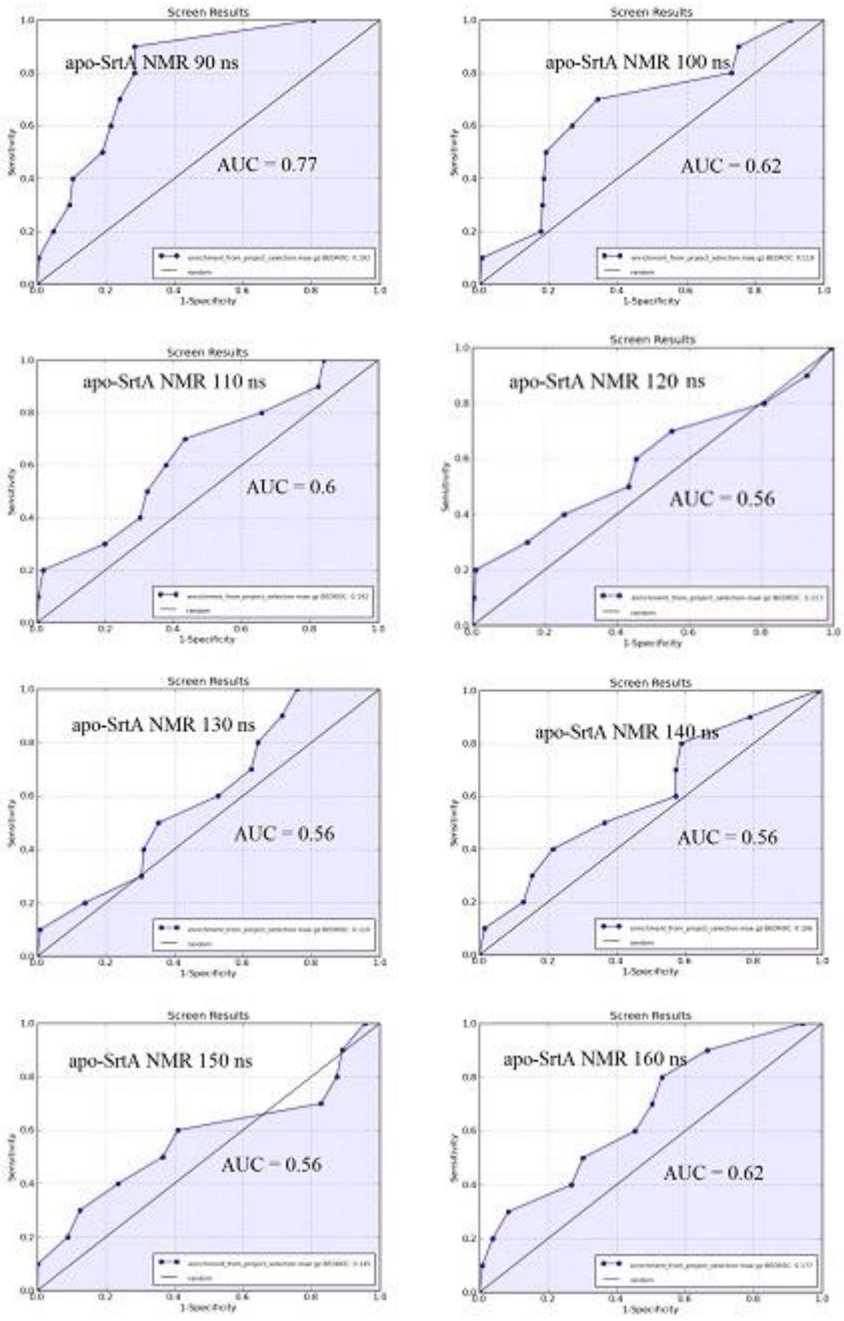
Screen Results
apo-SrtA NMR 90 ns
AUC = 0.77
Screen Results
apo-SrtA NMR 100 ns
AUC = 0.62
Screen Results
apo-SrtA NMR 110 ns
AUC = 0.6
Screen Results
apo-SrtA NMR 120 ns
AUC = 0.56
Screen Results
apo-SrtA NMR 130 ns
AUC = 0.56
Screen Results
apo-SrtA NMR 140 ns
AUC = 0.56
Screen Results
apo-SrtA NMR 150 ns
AUC = 0.56
Screen Results
apo-SrtA NMR 160 ns
AUC = 0.62
1-Specificity
Sensitivity

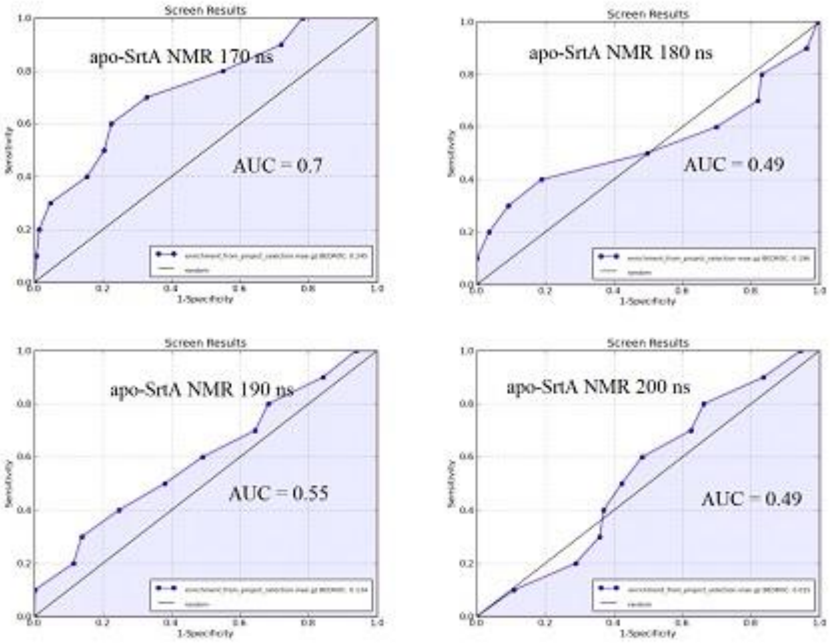

Screen Results
apo-SrtA NMR 170 ns
AUC = 0.7
Screen Results
apo-SrtA NMR 180 ns
AUC = 0.49
Screen Results
apo-SrtA NMR 190 ns
AUC = 0.55
Screen Results
apo-SrtA NMR 200 ns
AUC = 0.49
Sensitivity
1-Specificity

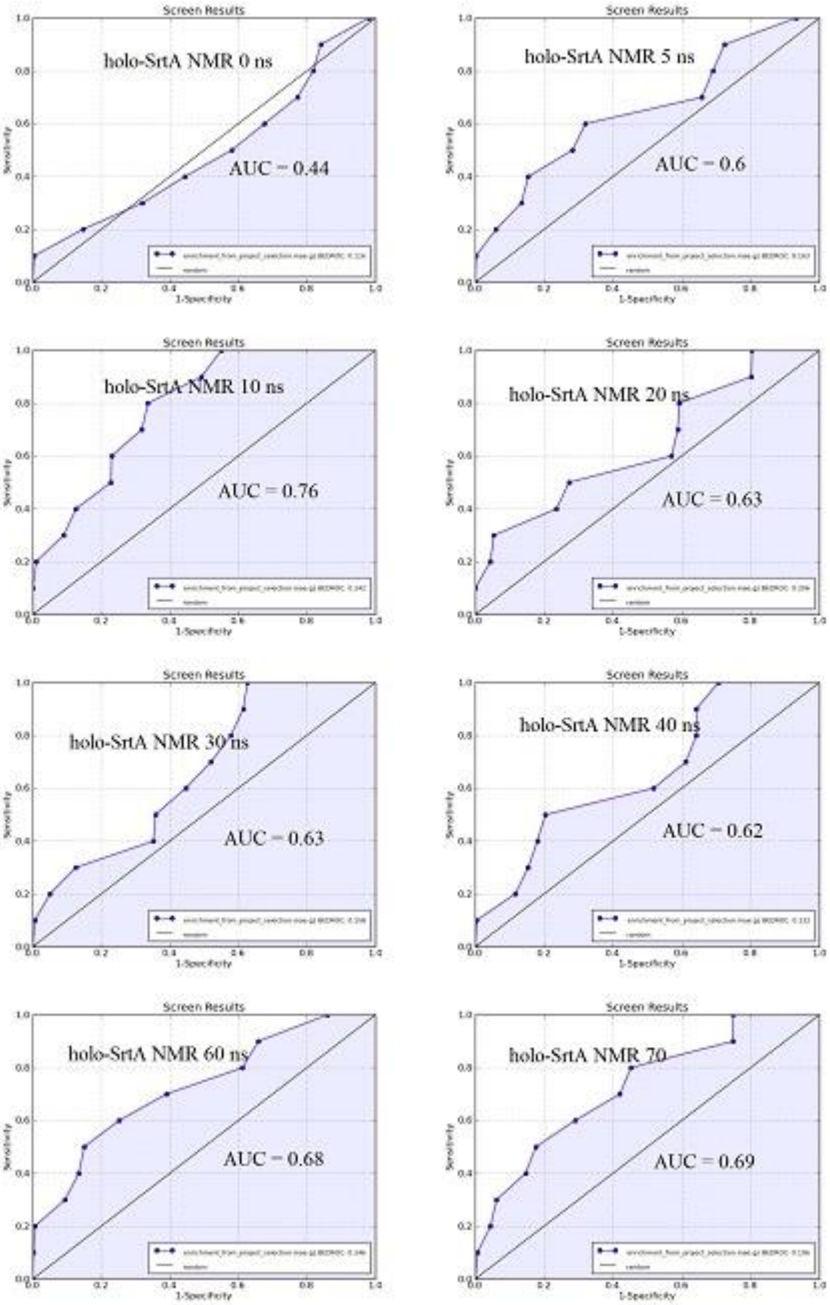
Screen Results
holo-SrtA NMR 0 ns
AUC = 0.44
Screen Results
holo-SrtA NMR 5 ns
AUC = 0.6
Screen Results
holo-SrtA NMR 10 ns
AUC = 0.76
Screen Results
holo-SrtA NMR 20 ns
AUC = 0.63
Screen Results
holo-SrtA NMR 30 ns
AUC = 0.63
Screen Results
holo-SrtA NMR 40 ns
AUC = 0.62
Screen Results
holo-SrtA NMR 60 ns
AUC = 0.68
Screen Results
holo-SrtA NMR 70
AUC = 0.69
1-Specificity
Sensitivity

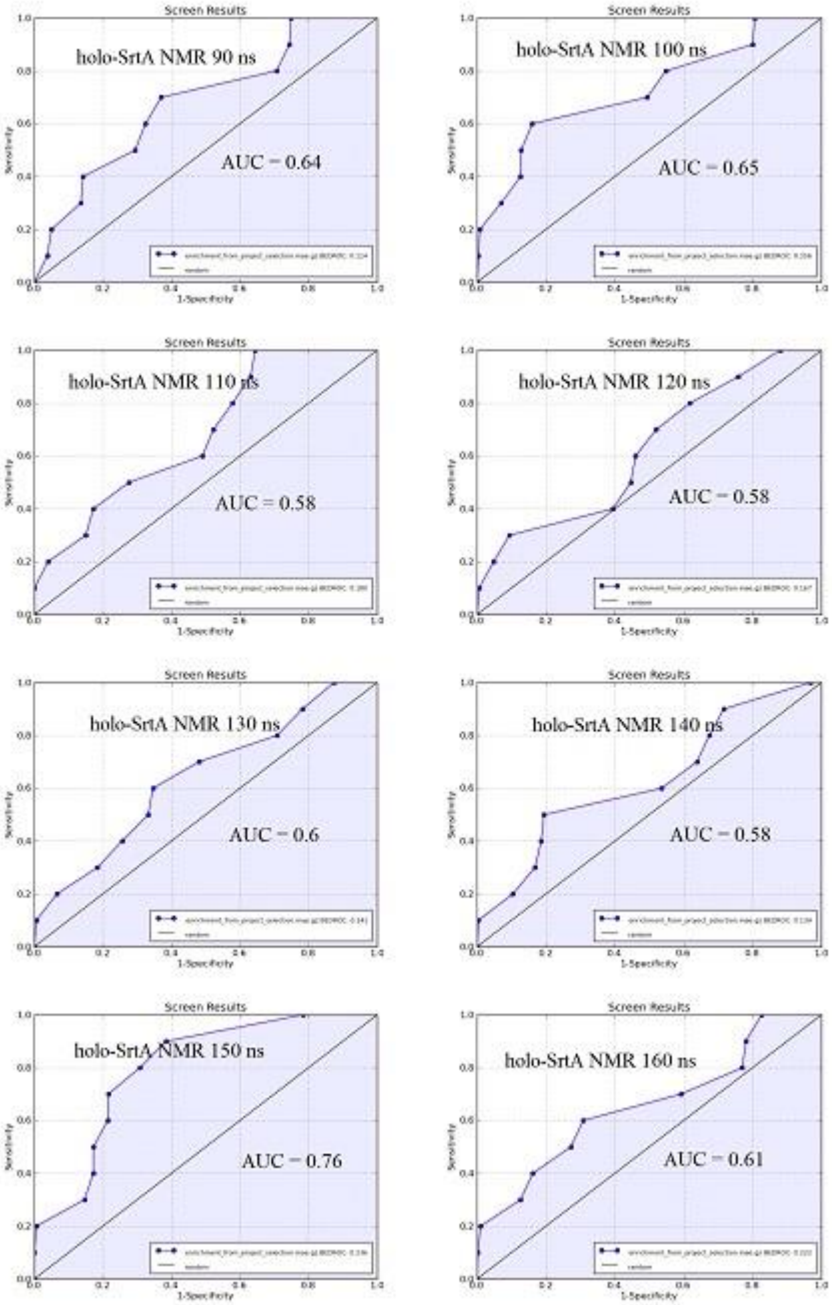
Screen Results
holo-SrtA NMR 90 ns
AUC = 0.64
Sensitivity
1-Specificity
Screen Results
holo-SrtA NMR 100 ns
AUC = 0.65
Sensitivity
1-Specificity
Screen Results
holo-SrtA NMR 110 ns
AUC = 0.58
Sensitivity
1-Specificity
Screen Results
holo-SrtA NMR 120 ns
AUC = 0.58
Sensitivity
1-Specificity
Screen Results
holo-SrtA NMR 130 ns
AUC = 0.6
Sensitivity
1-Specificity
Screen Results
holo-SrtA NMR 140 ns
AUC = 0.58
Sensitivity
1-Specificity
Screen Results
holo-SrtA NMR 150 ns
AUC = 0.76
Sensitivity
1-Specificity
Screen Results
holo-SrtA NMR 160 ns
AUC = 0.61
Sensitivity
1-Specificity

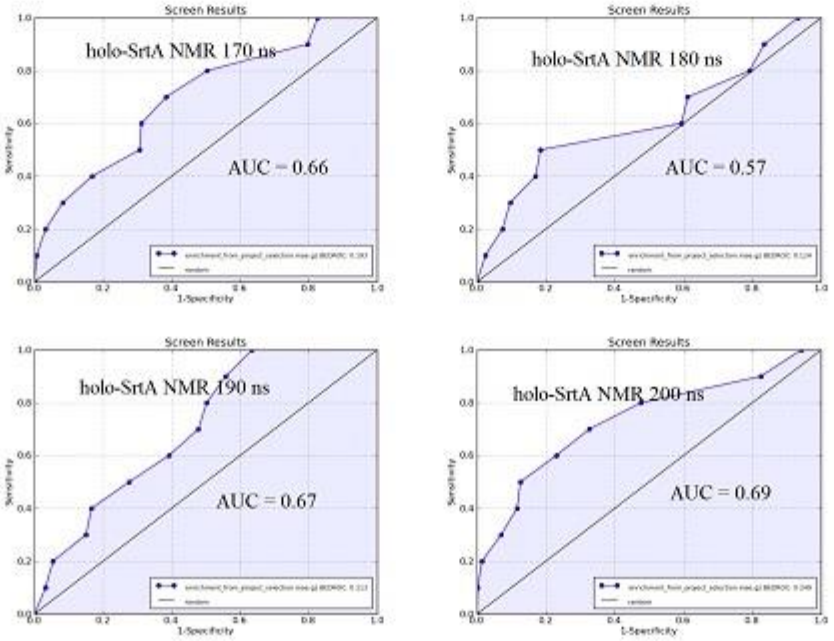

Screen Results
holo-SrtA NMR 170 ns
AUC = 0.66
Screen Results
holo-SrtA NMR 180 ns
AUC = 0.57
Screen Results
holo-SrtA NMR 190 ns
AUC = 0.67
Screen Results
holo-SrtA NMR 200 ns
AUC = 0.69
Sensitivity
1-Specificity

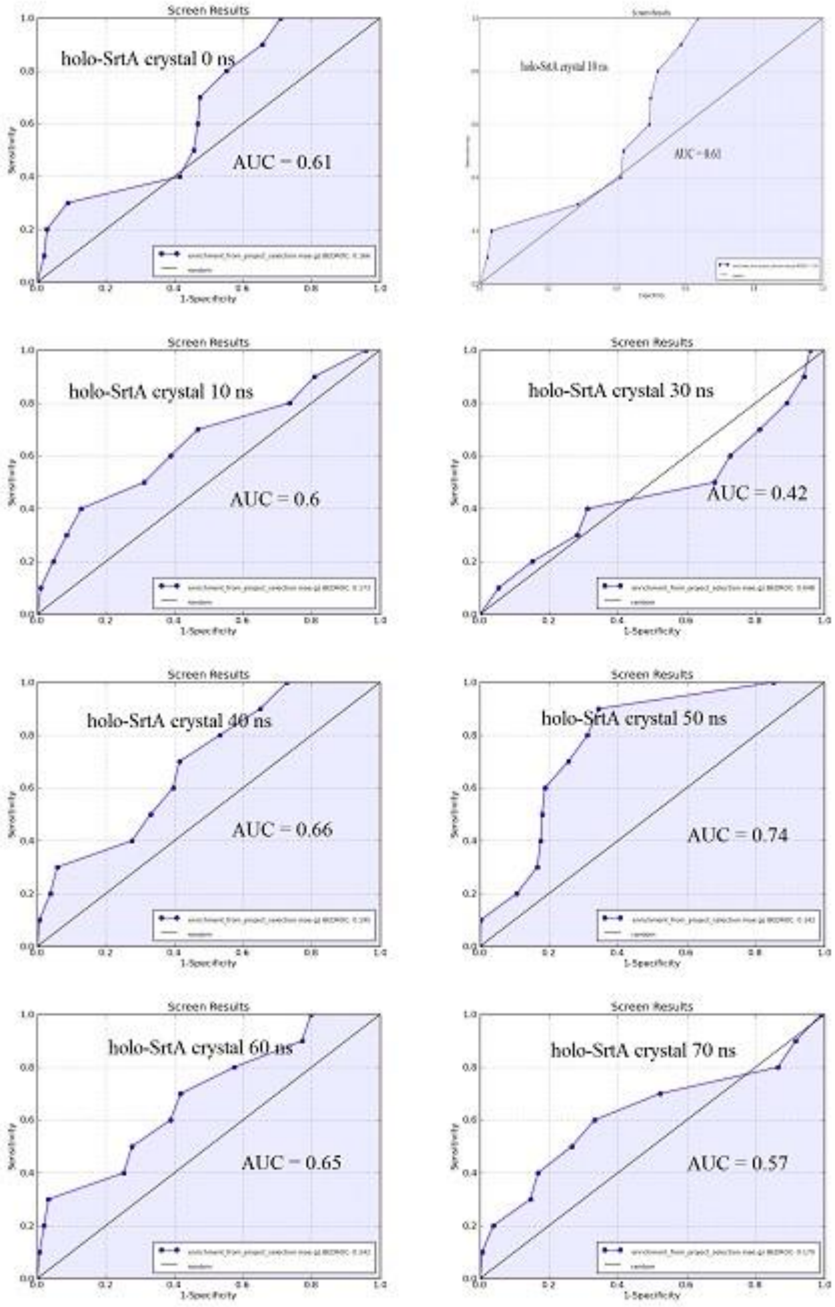

Screen Results
holo-SrtA crystal 0 ns
AUC = 0.61
Sensitivity
1-Specificity
holo-SrtA crystal 10 ns
AUC = 0.61
Screen Results
holo-SrtA crystal 10 ns
AUC = 0.6
Sensitivity
1-Specificity
Screen Results
holo-SrtA crystal 30 ns
AUC = 0.42
Sensitivity
1-Specificity
Screen Results
holo-SrtA crystal 40 ns
AUC = 0.66
Sensitivity
1-Specificity
Screen Results
holo-SrtA crystal 50 ns
AUC = 0.74
Sensitivity
1-Specificity
Screen Results
holo-SrtA crystal 60 ns
AUC = 0.65
Sensitivity
1-Specificity
Screen Results
holo-SrtA crystal 70 ns
AUC = 0.57
Sensitivity
1-Specificity

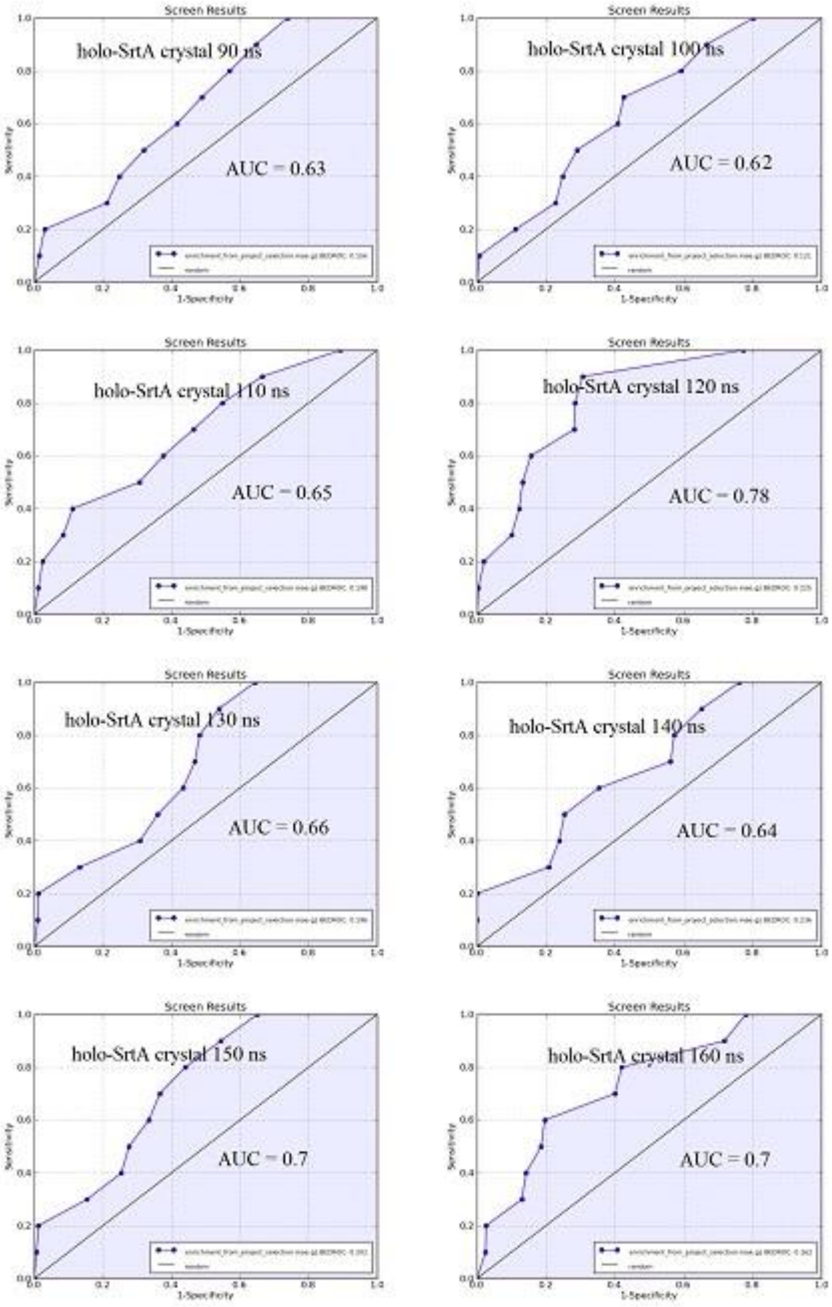
Screen Results
holo-SrtA crystal 90 ns
AUC = 0.63
Sensitivity
1-Specificity
Screen Results
holo-SrtA crystal 100 ns
AUC = 0.62
Sensitivity
1-Specificity
Screen Results
holo-SrtA crystal 110 ns
AUC = 0.65
Sensitivity
1-Specificity
Screen Results
holo-SrtA crystal 120 ns
AUC = 0.78
Sensitivity
1-Specificity
Screen Results
holo-SrtA crystal 130 ns
AUC = 0.66
Sensitivity
1-Specificity
Screen Results
holo-SrtA crystal 140 ns
AUC = 0.64
Sensitivity
1-Specificity
Screen Results
holo-SrtA crystal 150 ns
AUC = 0.7
Sensitivity
1-Specificity
Screen Results
holo-SrtA crystal 160 ns
AUC = 0.7
Sensitivity
1-Specificity

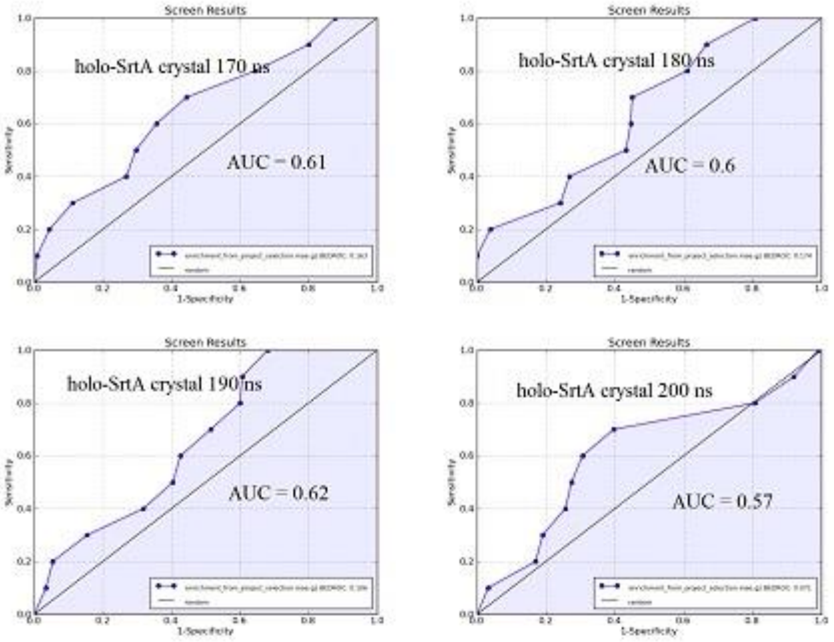
Screen Results
holo-SrtA crystal 170 ns
AUC = 0.61
Screen Results
holo-SrtA crystal 180 ns
AUC = 0.6
Screen Results
holo-SrtA crystal 190 ns
AUC = 0.62
Screen Results
holo-SrtA crystal 200 ns
AUC = 0.57
Sensitivity
1-Specificity

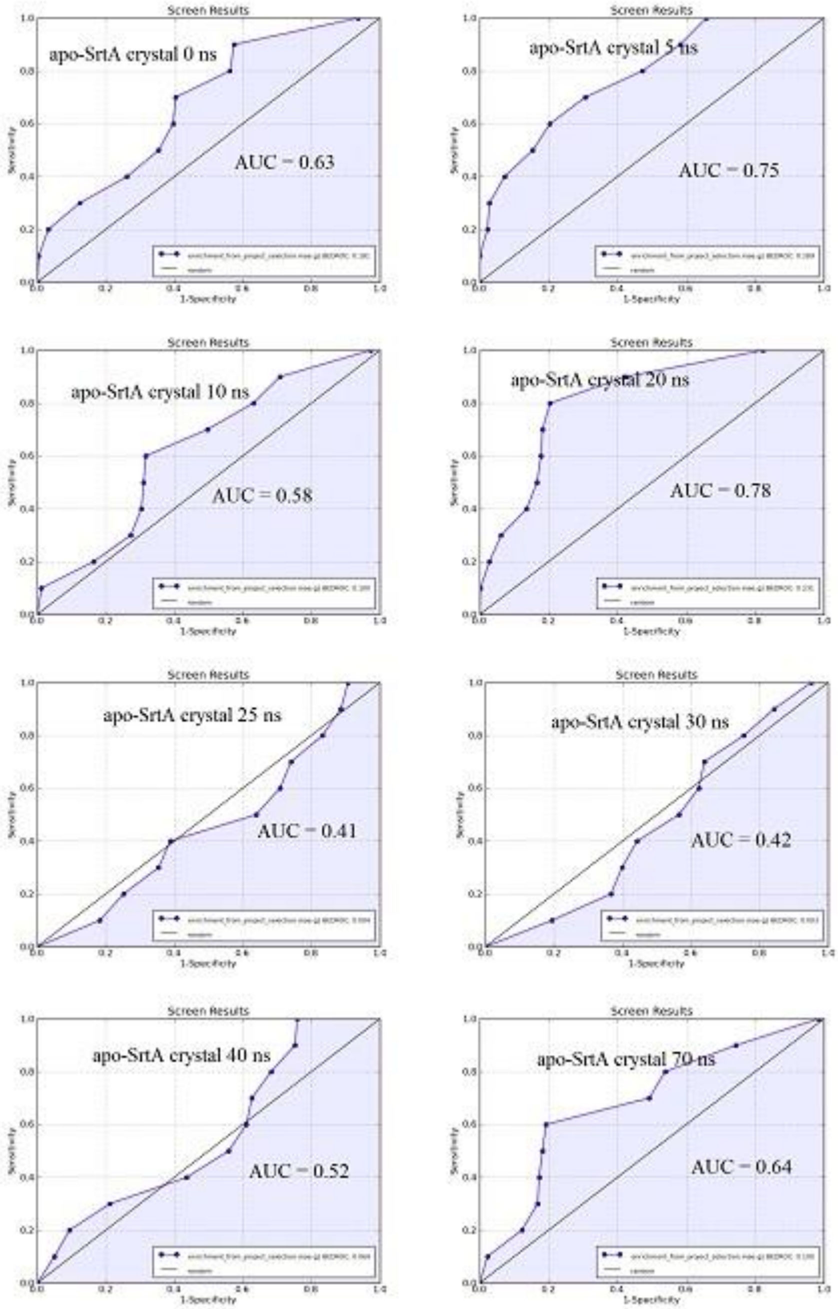
Screen Results
apo-SrtA crystal 0 ns
AUC = 0.63
Screen Results
apo-SrtA crystal 5 ns
AUC = 0.75
Screen Results
apo-SrtA crystal 10 ns
AUC = 0.58
Screen Results
apo-SrtA crystal 20 ns
AUC = 0.78
Screen Results
apo-SrtA crystal 25 ns
AUC = 0.41
Screen Results
apo-SrtA crystal 30 ns
AUC = 0.42
Screen Results
apo-SrtA crystal 40 ns
AUC = 0.52
Screen Results
apo-SrtA crystal 70 ns
AUC = 0.64
Sensitivity
1-Specificity

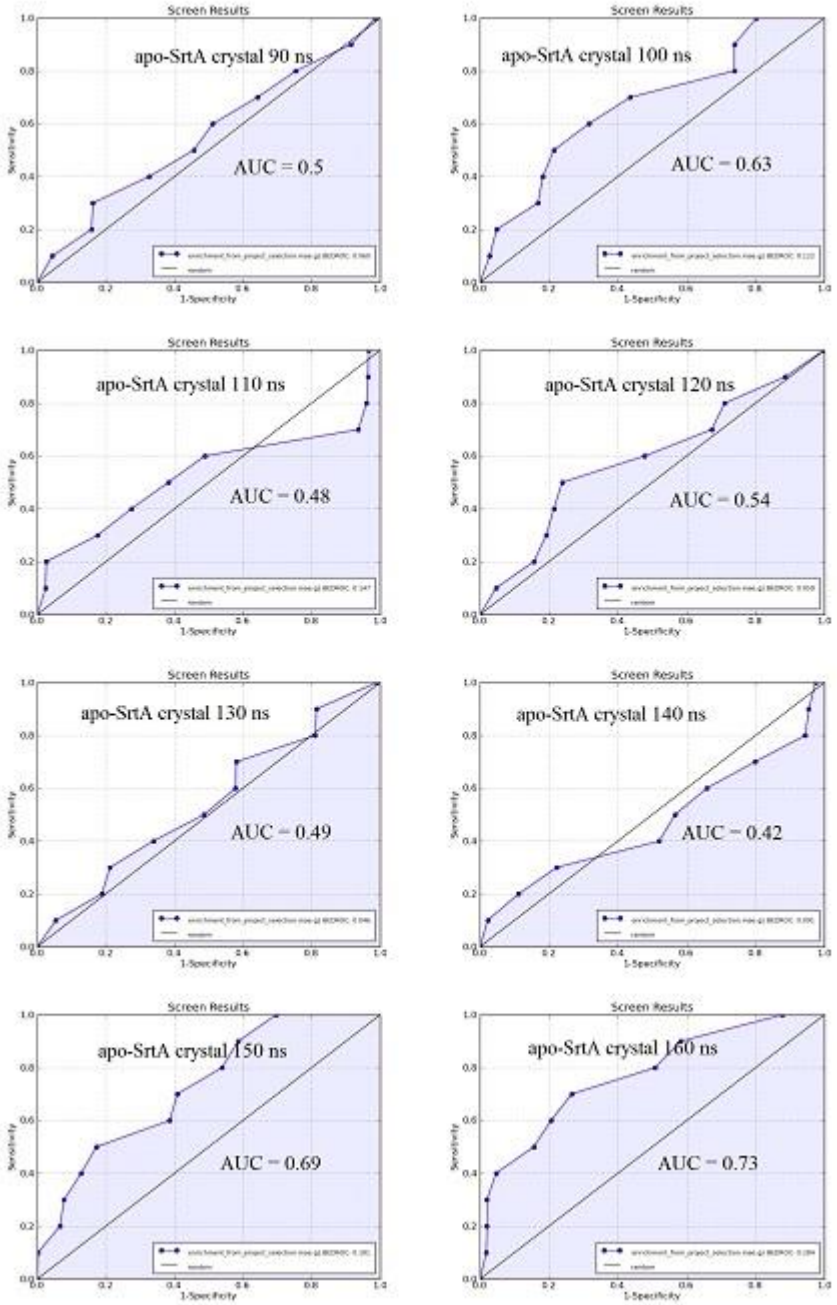
Screen Results
apo-SrtA crystal 90 ns
AUC = 0.5
1-Specificity
Screen Results
apo-SrtA crystal 100 ns
AUC = 0.63
1-Specificity
Screen Results
apo-SrtA crystal 110 ns
AUC = 0.48
1-Specificity
Screen Results
apo-SrtA crystal 120 ns
AUC = 0.54
1-Specificity
Screen Results
apo-SrtA crystal 130 ns
AUC = 0.49
1-Specificity
Screen Results
apo-SrtA crystal 140 ns
AUC = 0.42
1-Specificity
Screen Results
apo-SrtA crystal 150 ns
AUC = 0.69
1-Specificity
Screen Results
apo-SrtA crystal 160 ns
AUC = 0.73
1-Specificity

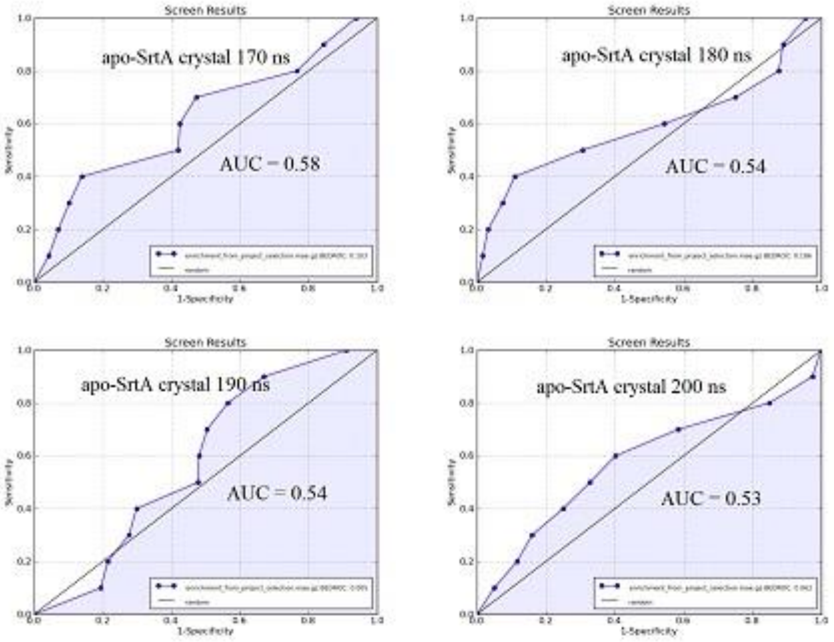

Figure S1. ROC plots for all the snapshots, for apo-SrtA NMR, holo-SrtA NMR, holo-SrtA Crystal structure, and apo-StrA Crystal structure, respectively..

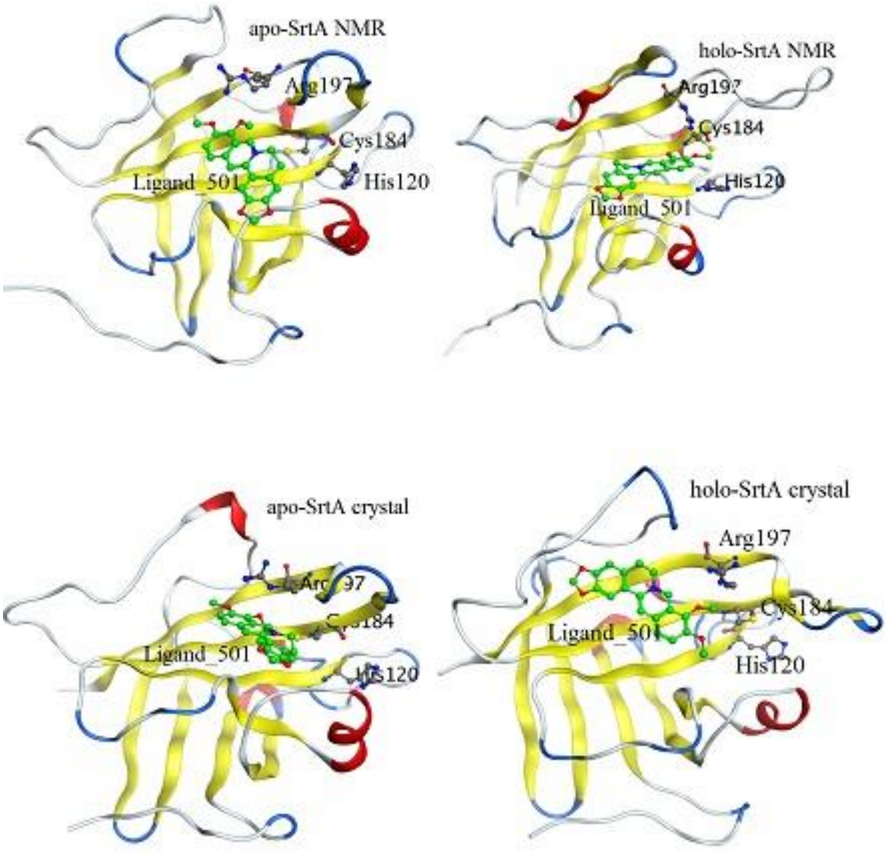
apo-SrtA NMR
Arg197
Cys184
Ligand_501
His120
holo-SrtA NMR
Arg197
Cys184
His120
ligand_501
apo-SrtA crystal
Ligand_501
His120
holo-SrtA crystal
Arg197
Cys184
Ligand_501
His120

(Ligand_501)

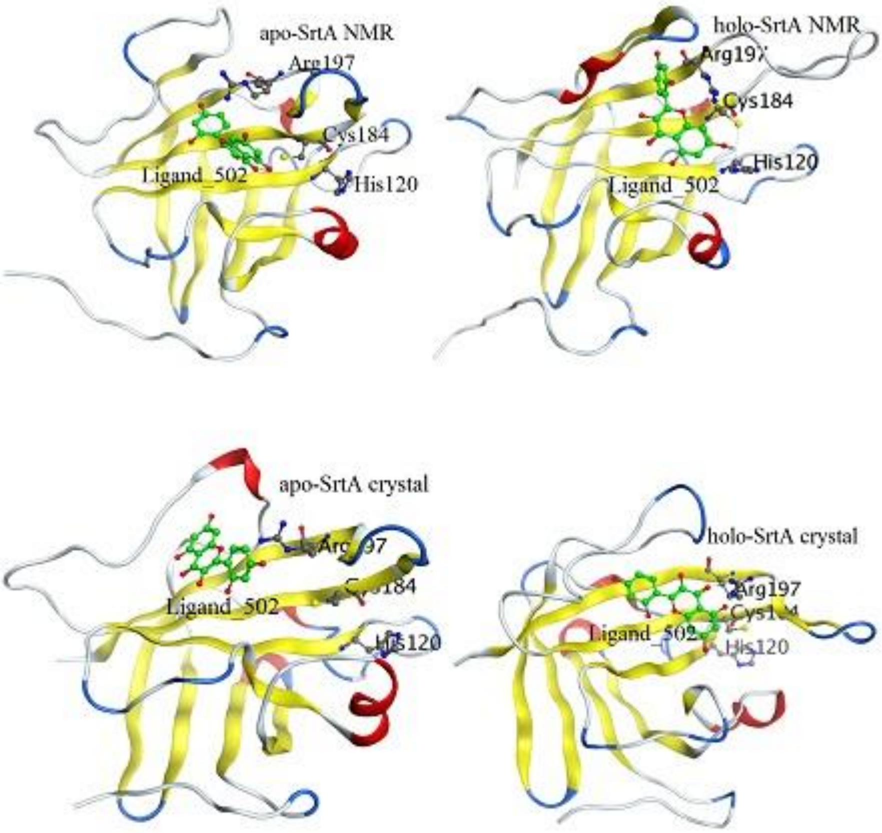
apo-SrtA NMR
Arg197
Cys184
Ligand_502
His120
holo-SrtA NMR
Cys184
His120
Ligand_502
apo-SrtA crystal
Ligand_502
His120
holo-SrtA crystal
Arg197
Ligand_502
His120

(Ligand_502)

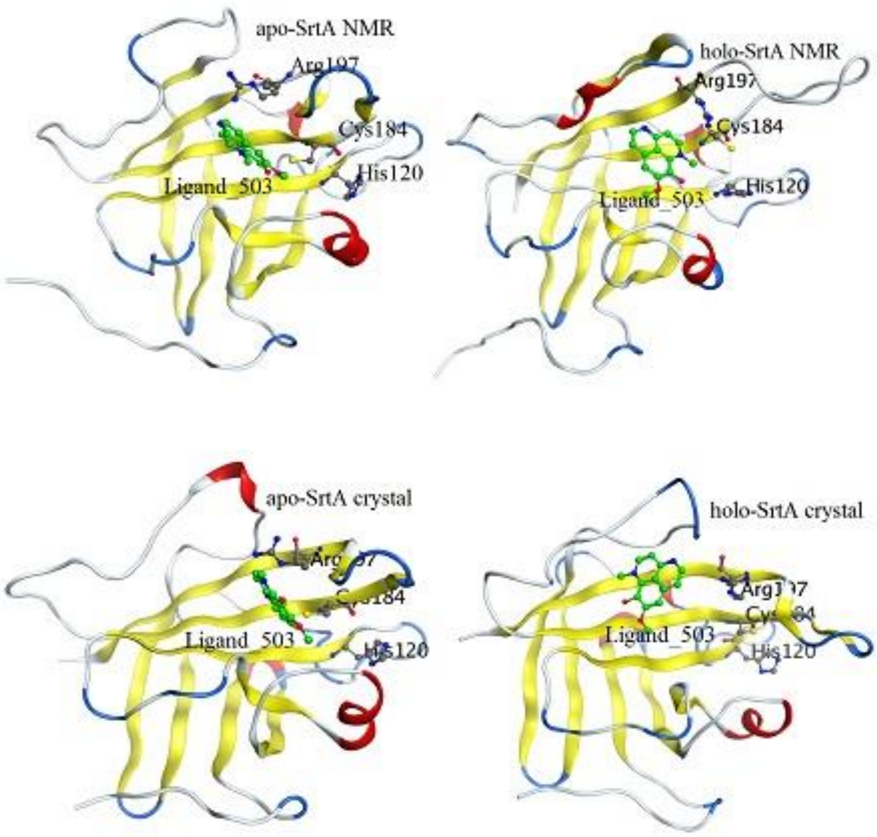
apo-SrtA NMR
Arg197
Cys184
Ligand_503
His120
holo-SrtA NMR
Arg197
Cys184
Ligand_503
His120
apo-SrtA crystal
Ligand_503
holo-SrtA crystal
Ligand_503
His120

(Ligand_503)

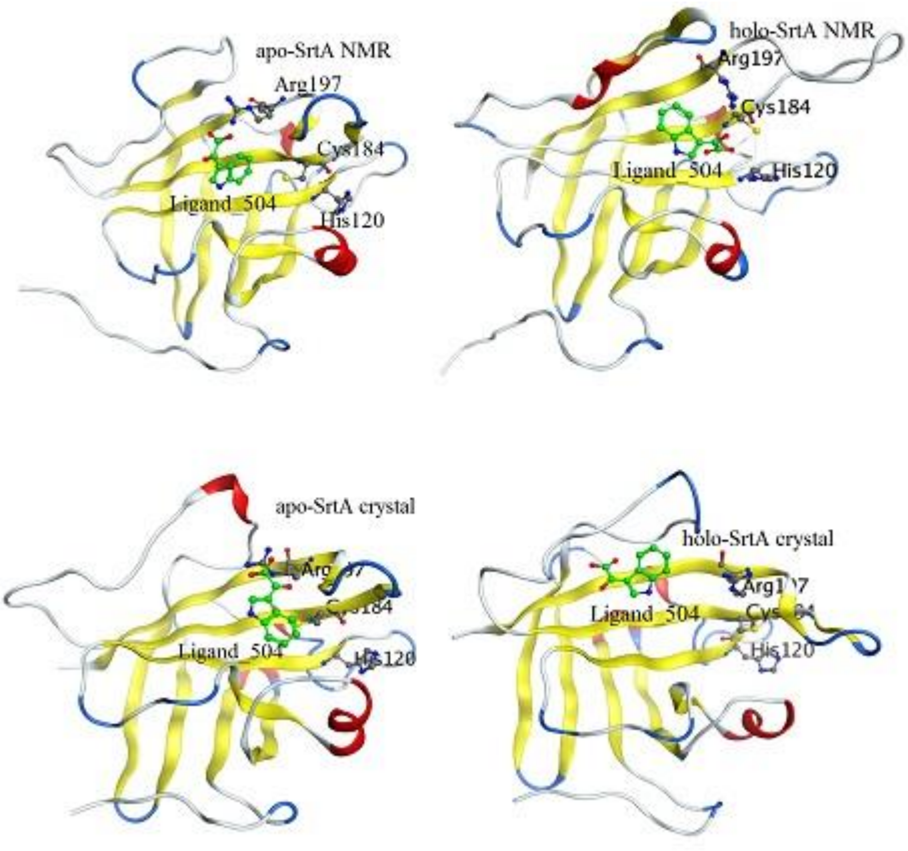
apo-SrtA NMR
Arg197
Cys184
Ligand_504
His120
holo-SrtA NMR
Arg197
Cys184
Ligand_504
His120
apo-SrtA crystal
Ligand_504
holo-SrtA crystal
Ligand_504
His120

(Ligand_504)

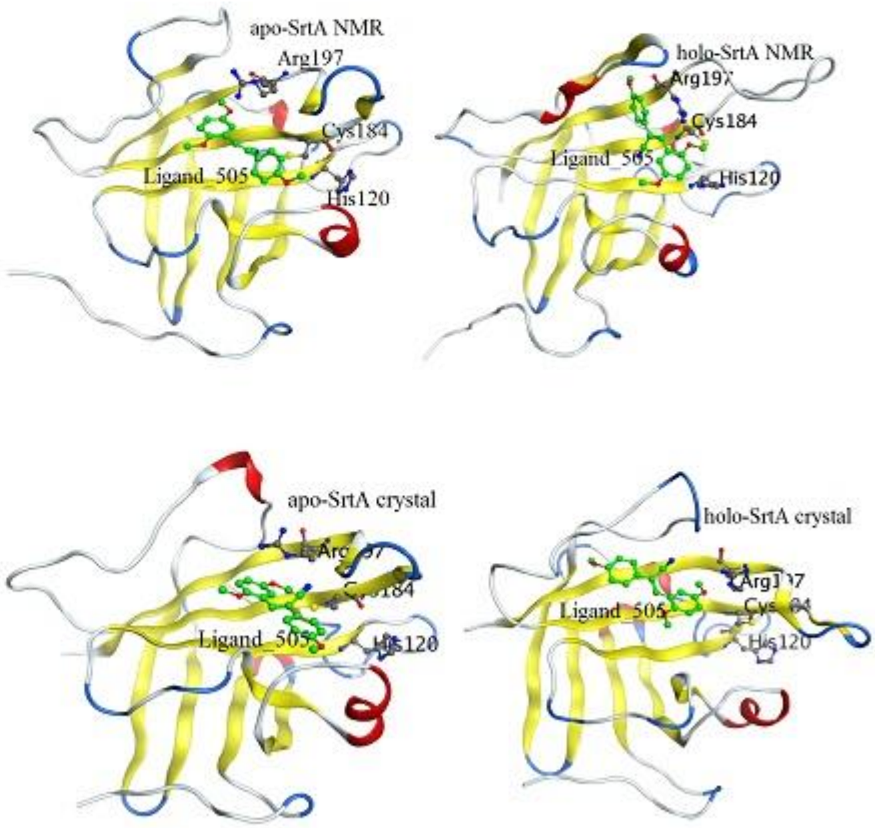
apo-SrtA NMR
Arg197
Cys184
Ligand_505
His120
holo-SrtA NMR
Arg197
Cys184
Ligand_505
His120
apo-SrtA crystal
Ligand_505
His120
holo-SrtA crystal
Ligand_505
His120

(Ligand_505)

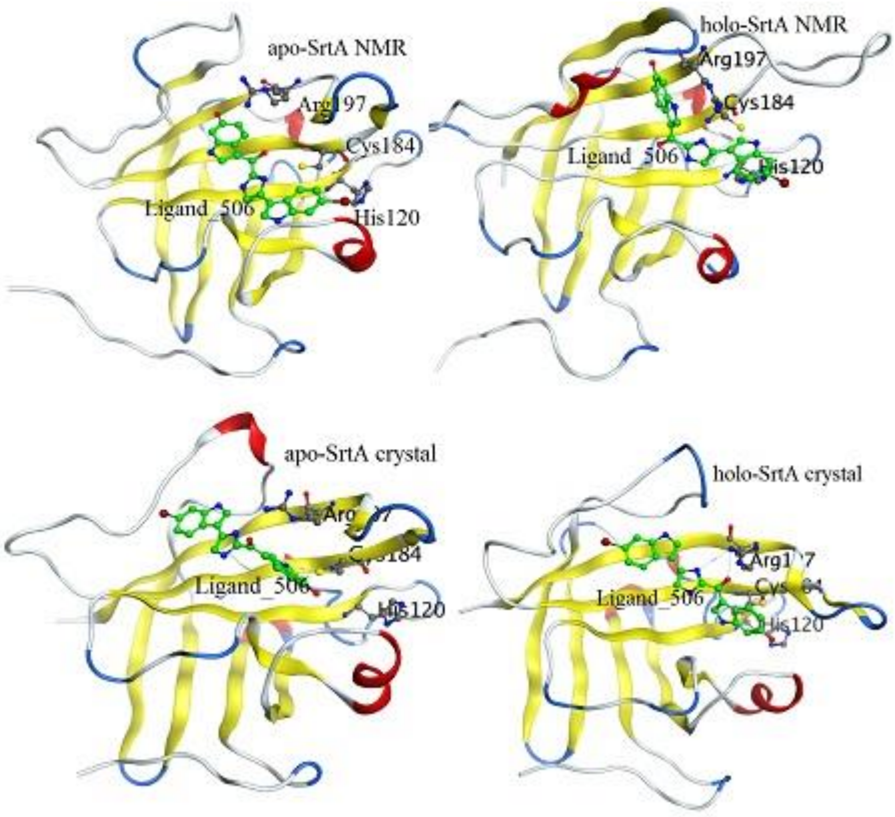
apo-SrtA NMR
Arg197
Cys184
Ligand_506
His120
holo-SrtA NMR
Arg197
Cys184
Ligand_506
His120
apo-SrtA crystal
Cys184
Ligand_506
His120
holo-SrtA crystal
Ligand_506
His120

(Ligand_506)

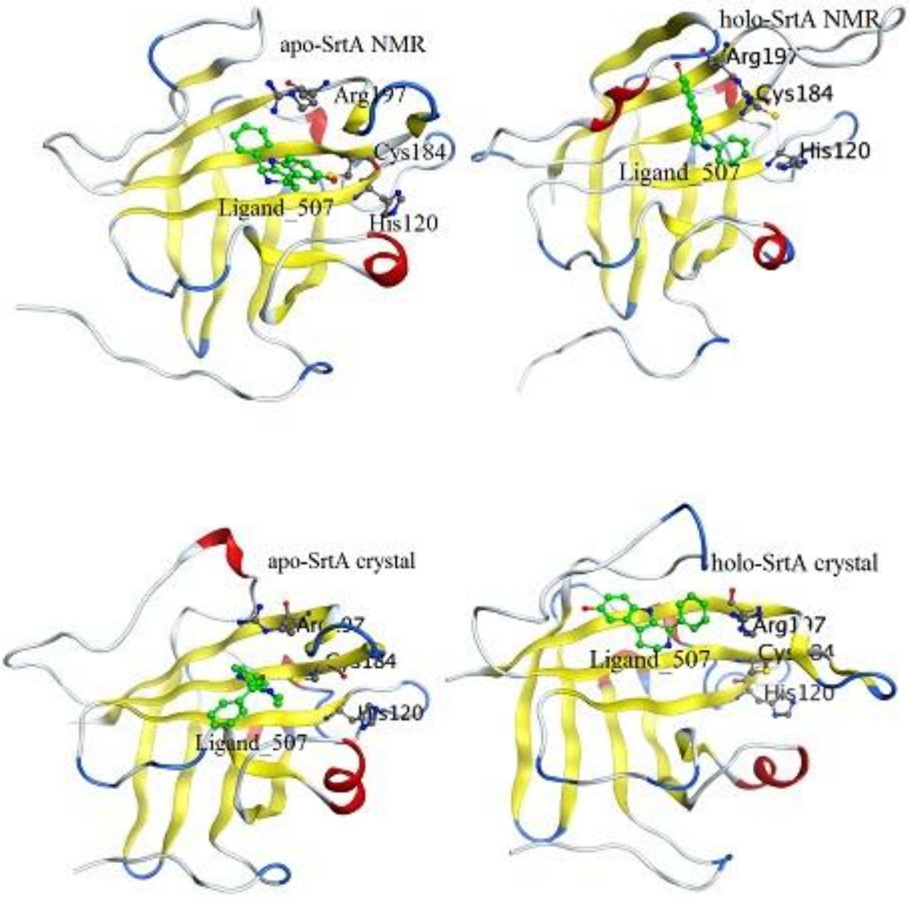

apo-SrtA NMR
Arg197
Cys184
Ligand_507
His120
holo-SrtA NMR
Cys184
His120
Ligand_507
apo-SrtA crystal
Ligand_507
holo-SrtA crystal
Ligand_507
His120

(Ligand_507)

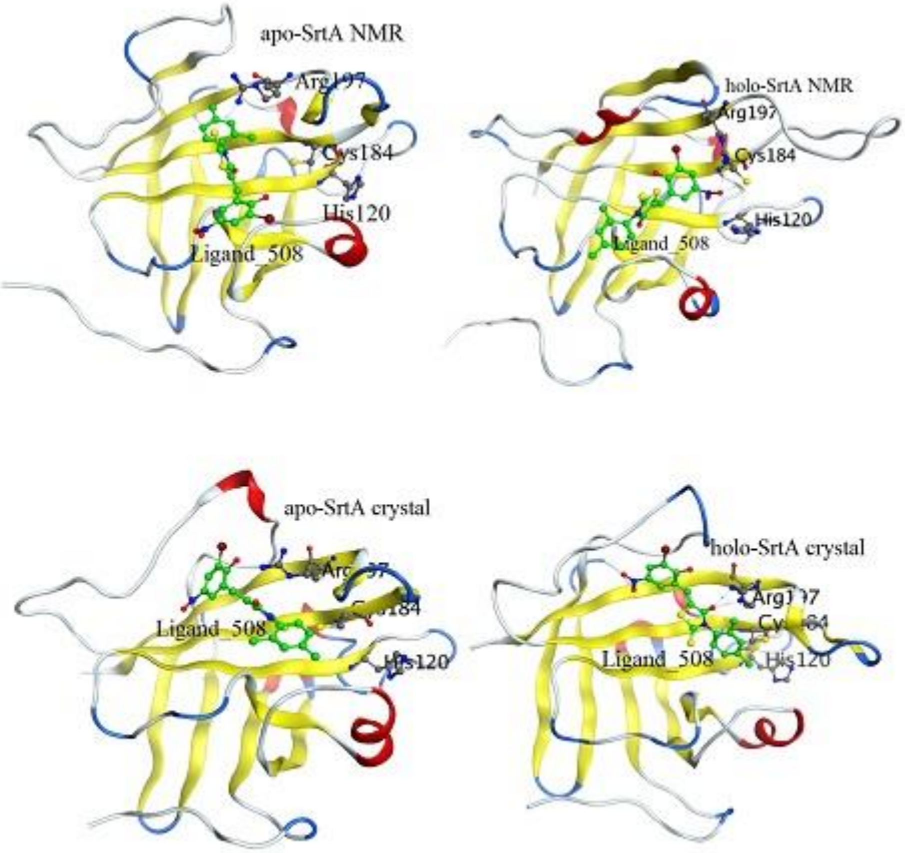
apo-SrtA NMR
Arg197
Cys184
His120
Ligand_508
holo-SrtA NMR
Arg197
Cys184
His120
apo-SrtA crystal
Ligand_508
holo-SrtA crystal
Ligand_508
His120

(Ligand_508)

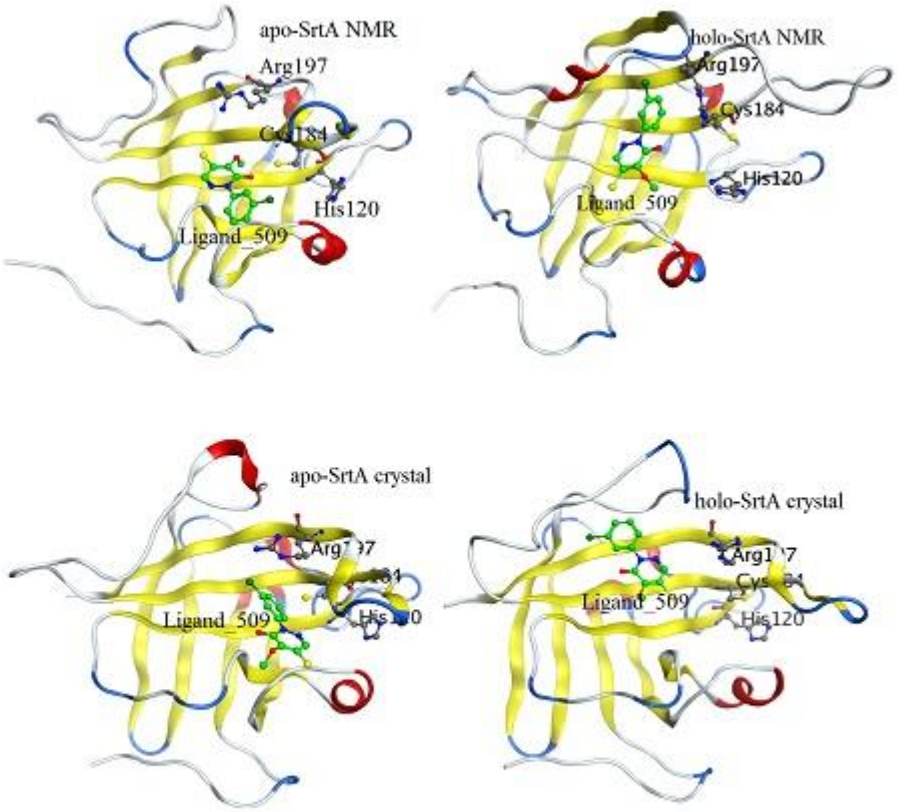
apo-SrtA NMR
Arg197
His120
Ligand_509
holo-SrtA NMR
Arg197
Cys184
His120
Ligand_509
apo-SrtA crystal
Ligand_509
holo-SrtA crystal
Ligand_509
His120

(Ligand_509)

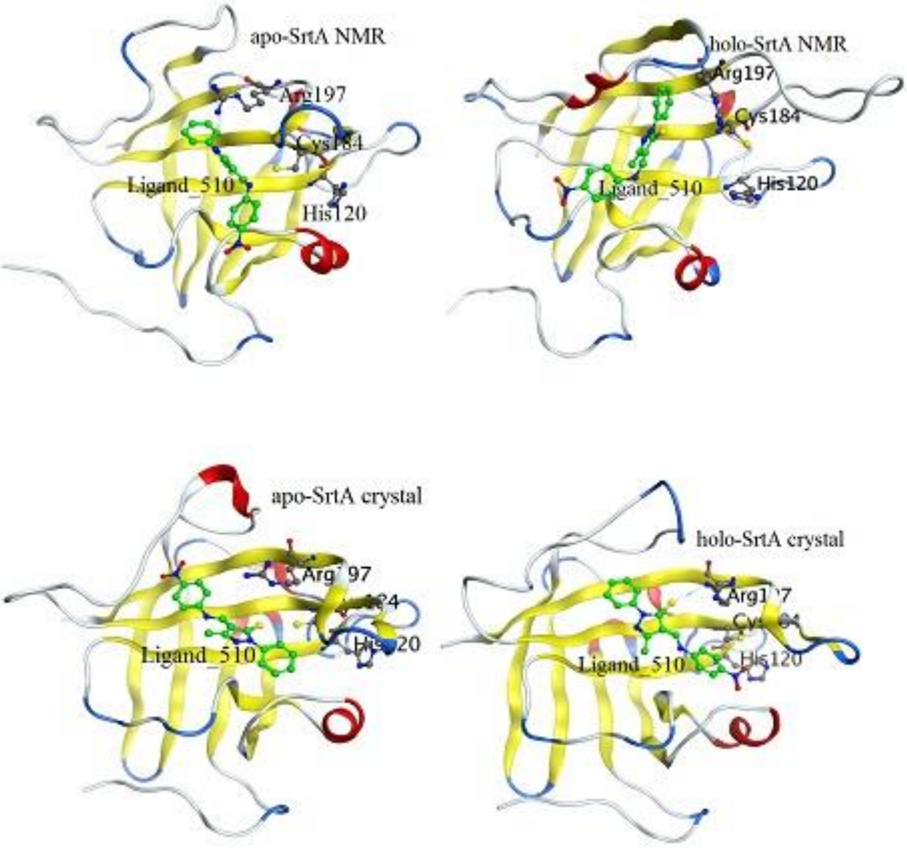

(Ligand_510)

Figure S2. Docking poses for ligands 501 – 510, for all four receptor structures in the initial snapshot.

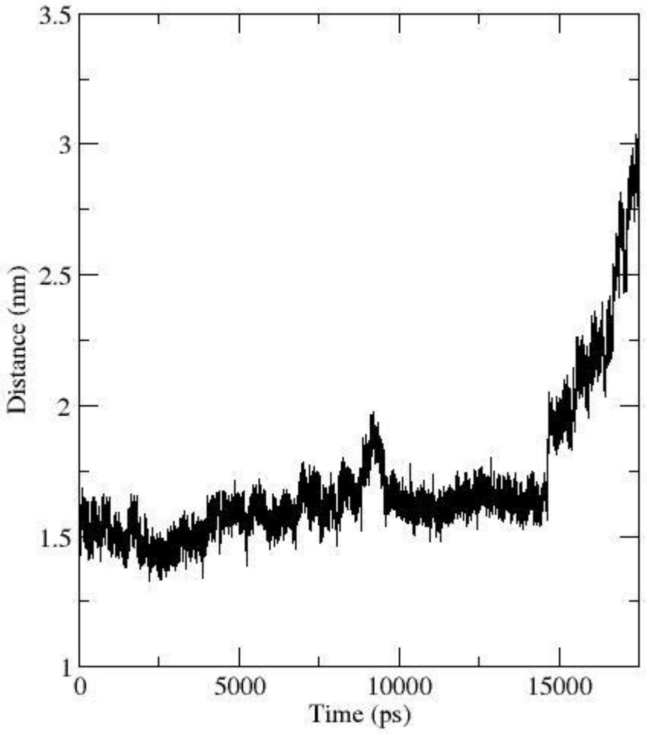

Figure S3. Distance between V166-V169 of the β6/β7 loop and C184, monitored in the SMD pulling process.

Table S1. Data for Figure 5.

	rmsd	distance	$EF^{1\%}$	AUC	RIE	BEDROC(α=160.9)	BEDROC(α=20)
apo-SrtA NMR structure	4,26	13,28	8,5	0,57	2,68	0,155	0,162
Holo-SrtA NMR structure	3,61	14,71	9	0,64	2,97	0,172	0,179
apo-SrtA crystal structure	4,63	20,38	2	0,57	1,92	0,049	0,116
Holo-SrtA crystal structure	2,87	17,39	5,5	0,63	2,68	0,1	0,162

Table S2. Ligand ranking for apo-SrtA NMR structure

MD snapshot (ns)	0		5		10		20		40	
	Rank	Ligand	Rank	Ligand	Rank	ligand	Rank	Ligand	Rank	Ligand
	2	ligand_502	11	ligand_502	1	ligand_502	8	ligand_502	5	ligand_502
	30	ligand_506	52	ligand_503	4	ligand_504	73	ligand_504	48	ligand_509
	40	ligand_504	95	ligand_504	35	ligand_503	261	ligand_503	144	ligand_508
	96	ligand_509	190	ligand_501	209	ligand_507	262	ligand_507	176	ligand_504
	230	ligand_503	260	ligand_508	229	ligand_509	310	ligand_509	220	ligand_506
	232	ligand_507	296	ligand_509	366	ligand_506	357	ligand_506	321	ligand_503
	272	ligand_505	328	ligand_505	453	ligand_508	387	ligand_508	328	ligand_505
	330	ligand_510	385	ligand_510	458	ligand_510	475	ligand_510	330	ligand_507
	413	ligand_508	394	ligand_506	480	ligand_505	477	ligand_505	476	ligand_510
	505	ligand_501	466	ligand_507	506	ligand_501	506	ligand_501	502	ligand_501
	60		70		80		90		100	
	Rank	Ligand	Rank	Ligand	Rank	ligand	Rank	Ligand	Rank	Ligand
	2	ligand_502	2	ligand_502	1	ligand_502	3	ligand_502	4	ligand_502
	8	ligand_506	15	ligand_504	33	ligand_506	25	ligand_503	91	ligand_504
	40	ligand_504	25	ligand_509	76	ligand_509	50	ligand_508	94	ligand_506
	110	ligand_509	53	ligand_506	119	ligand_507	56	ligand_504	97	ligand_508
	207	ligand_503	108	ligand_503	231	ligand_504	100	ligand_509	101	ligand_503
	295	ligand_507	109	ligand_508	291	ligand_508	113	ligand_505	140	ligand_507
	432	ligand_510	115	ligand_505	293	ligand_503	127	ligand_501	178	ligand_509
	436	ligand_505	128	ligand_507	305	ligand_505	150	ligand_507	373	ligand_510
	439	ligand_501	230	ligand_510	338	ligand_510	151	ligand_506	385	ligand_501
	472	ligand_508	276	ligand_501	382	ligand_501	413	ligand_510	462	ligand_505
	110		120		130		140		150	
	Rank	Ligand	Rank	Ligand	Rank	ligand	Rank	Ligand	Rank	Ligand
	1	ligand_502	3	ligand_506	5	ligand_502	8	ligand_502	2	ligand_502
	3	ligand_504	6	ligand_502	71	ligand_506	66	ligand_509	46	ligand_503
	11	ligand_510	79	ligand_504	155	ligand_503	80	ligand_504	65	ligand_507
	102	ligand_503	131	ligand_505	159	ligand_504	111	ligand_506	122	ligand_510
	155	ligand_505	221	ligand_508	182	ligand_505	187	ligand_501	188	ligand_505
	166	ligand_506	233	ligand_509	270	ligand_508	292	ligand_503	211	ligand_506
	195	ligand_509	283	ligand_503	319	ligand_507	293	ligand_505	421	ligand_509
	224	ligand_508	412	ligand_507	330	ligand_509	302	ligand_507	445	ligand_504
	337	ligand_501	472	ligand_510	366	ligand_510	403	ligand_508	454	ligand_508
	421	ligand_507	507	ligand_501	389	ligand_501	503	ligand_510	488	ligand_501

160		170		180		190		200	
Rank	Ligand	Rank	Ligand	Rank	ligand	Rank	Ligand	Rank	Ligand
5	ligand_502	4	ligand_502	1	ligand_502	2	ligand_502	55	ligand_509
21	ligand_503	9	ligand_504	20	ligand_506	59	ligand_509	146	ligand_503
45	ligand_504	26	ligand_509	49	ligand_509	72	ligand_504	182	ligand_510
138	ligand_507	81	ligand_503	98	ligand_503	127	ligand_506	189	ligand_508
156	ligand_506	107	ligand_506	253	ligand_504	195	ligand_507	216	ligand_502
233	ligand_508	118	ligand_505	355	ligand_510	251	ligand_510	247	ligand_506
259	ligand_510	171	ligand_510	417	ligand_507	329	ligand_508	319	ligand_507
274	ligand_509	283	ligand_501	423	ligand_505	349	ligand_503	339	ligand_501
341	ligand_505	369	ligand_508	490	ligand_501	430	ligand_505	427	ligand_505
481	ligand_501	401	ligand_507	507	ligand_508	479	ligand_501	482	ligand_504

Table S3. Ligand ranking for holo-SrtA NMR structure

MD snapshot (ns)	0		5		10		20		30	
	Rank	Ligand	Rank	Ligand	Rank	Ligand	Rank	Ligand	Rank	ligand
	3	ligand_502	2	ligand_502	1	ligand_502	1	ligand_506	4	ligand_502
	75	ligand_507	32	ligand_506	7	ligand_506	24	ligand_502	27	ligand_506
	163	ligand_506	70	ligand_504	48	ligand_509	30	ligand_505	66	ligand_504
	226	ligand_505	81	ligand_507	67	ligand_507	122	ligand_510	180	ligand_503
	295	ligand_509	146	ligand_505	119	ligand_505	141	ligand_507	184	ligand_505
	344	ligand_504	166	ligand_509	121	ligand_503	291	ligand_509	229	ligand_510
	393	ligand_503	336	ligand_501	166	ligand_508	301	ligand_504	267	ligand_501
	417	ligand_510	353	ligand_510	176	ligand_510	304	ligand_501	297	ligand_508
	429	ligand_501	371	ligand_503	255	ligand_501	410	ligand_508	316	ligand_507
	500	ligand_508	476	ligand_508	285	ligand_504	412	ligand_503	323	ligand_509

	40		60		70		90		100	
	Rank	Ligand	Rank	Ligand	Rank	Ligand	Rank	Ligand	Rank	Ligand
	3	ligand_502	2	ligand_502	4	ligand_502	14	ligand_502	20	ligand_506
	60	ligand_504	5	ligand_506	24	ligand_506	35	ligand_503	27	ligand_502
	80	ligand_506	50	ligand_508	34	ligand_509	133	ligand_507	71	ligand_501
	94	ligand_509	71	ligand_504	77	ligand_504	174	ligand_510	75	ligand_503
	107	ligand_507	80	ligand_503	93	ligand_507	186	ligand_504	152	ligand_508
	265	ligand_503	132	ligand_505	151	ligand_503	205	ligand_506	168	ligand_504
	313	ligand_505	202	ligand_509	217	ligand_508	324	ligand_509	192	ligand_509
	329	ligand_510	314	ligand_507	234	ligand_510	363	ligand_505	361	ligand_505
	330	ligand_501	338	ligand_501	383	ligand_501	483	ligand_508	381	ligand_507
	363	ligand_508	440	ligand_510	384	ligand_505	487	ligand_501	384	ligand_510

	110		120		130		140		150	
	Rank	Ligand	Rank	Ligand	Rank	Ligand	Rank	Ligand	Rank	Ligand
	3	ligand_507	1	ligand_502	4	ligand_506	4	ligand_502	3	ligand_502
	6	ligand_506	22	ligand_506	26	ligand_505	35	ligand_506	54	ligand_506
	38	ligand_503	78	ligand_505	50	ligand_502	95	ligand_504	87	ligand_504
	67	ligand_502	90	ligand_507	201	ligand_501	132	ligand_501	97	ligand_509
	69	ligand_501	143	ligand_508	228	ligand_504	171	ligand_503	102	ligand_507
	86	ligand_504	251	ligand_501	236	ligand_510	179	ligand_505	274	ligand_503
	254	ligand_505	268	ligand_504	267	ligand_507	247	ligand_510	326	ligand_501
	282	ligand_508	297	ligand_503	317	ligand_509	362	ligand_508	345	ligand_505
	409	ligand_510	324	ligand_510	388	ligand_503	400	ligand_509	367	ligand_508
	413	ligand_509	332	ligand_509	450	ligand_508	447	ligand_507	494	ligand_510

160		170		180		190		200	
Rank	Ligand	Rank	Ligand	Rank	Ligand	Rank	Ligand	Rank	Ligand
1	ligand_502	4	ligand_502	13	ligand_506	17	ligand_502	2	ligand_506
5	ligand_506	18	ligand_506	39	ligand_503	29	ligand_506	9	ligand_507
76	ligand_503	44	ligand_509	51	ligand_502	78	ligand_501	38	ligand_503
90	ligand_505	88	ligand_507	89	ligand_504	87	ligand_504	62	ligand_502
91	ligand_507	158	ligand_510	97	ligand_510	143	ligand_503	68	ligand_501
113	ligand_508	162	ligand_503	303	ligand_508	202	ligand_509	121	ligand_504
115	ligand_509	199	ligand_508	313	ligand_505	246	ligand_508	170	ligand_505
162	ligand_504	260	ligand_504	404	ligand_507	259	ligand_505	246	ligand_508
201	ligand_501	407	ligand_505	426	ligand_509	288	ligand_510	421	ligand_509
402	ligand_510	423	ligand_501	476	ligand_501	327	ligand_507	481	ligand_510

Table S4. Ligand ranking for apo-SrtA crystal structure

MD snapshot (ns)	0		5		10		20		25	
	Rank	Ligand	Rank	Ligand	Rank	ligand	Rank	Ligand	Rank	Ligand
	3	ligand_502	1	ligand_502	7	ligand_502	2	ligand_502	92	ligand_503
	18	ligand_506	13	ligand_506	84	ligand_507	16	ligand_506	128	ligand_506
	65	ligand_503	17	ligand_504	139	ligand_509	34	ligand_504	180	ligand_510
	135	ligand_509	40	ligand_508	156	ligand_504	72	ligand_507	198	ligand_504
	182	ligand_505	82	ligand_507	160	ligand_503	88	ligand_503	324	ligand_502
	204	ligand_501	108	ligand_510	164	ligand_506	95	ligand_501	360	ligand_507
	209	ligand_508	160	ligand_505	255	ligand_508	98	ligand_508	377	ligand_508
	289	ligand_510	244	ligand_509	323	ligand_501	110	ligand_505	424	ligand_505
	296	ligand_504	300	ligand_501	363	ligand_505	221	ligand_509	452	ligand_501
	478	ligand_507	339	ligand_503	496	ligand_510	421	ligand_510	463	ligand_509
	30		40		70		90		100	
	Rank	Ligand	Rank	Ligand	Rank	ligand	Rank	Ligand	Rank	Ligand
	98	ligand_504	26	ligand_502	12	ligand_502	23	ligand_504	15	ligand_502
	185	ligand_502	49	ligand_504	63	ligand_503	81	ligand_509	26	ligand_507
	202	ligand_507	109	ligand_501	87	ligand_508	84	ligand_502	87	ligand_504
	225	ligand_509	222	ligand_509	90	ligand_510	167	ligand_505	95	ligand_506
	287	ligand_503	284	ligand_505	96	ligand_506	233	ligand_503	112	ligand_501
	317	ligand_510	311	ligand_506	102	ligand_504	262	ligand_506	164	ligand_510
	326	ligand_506	320	ligand_510	253	ligand_509	328	ligand_510	225	ligand_505
	385	ligand_508	349	ligand_508	277	ligand_507	385	ligand_507	377	ligand_508
	430	ligand_505	385	ligand_507	381	ligand_505	466	ligand_508	378	ligand_503
	485	ligand_501	389	ligand_503	502	ligand_501	503	ligand_501	411	ligand_509
	110		120		130		140		150	
	Rank	Ligand	Rank	Ligand	Rank	ligand	Rank	Ligand	Rank	Ligand
	13	ligand_502	24	ligand_502	28	ligand_506	12	ligand_504	2	ligand_502
	15	ligand_504	80	ligand_503	96	ligand_502	58	ligand_502	35	ligand_507
	91	ligand_503	99	ligand_506	109	ligand_509	114	ligand_507	42	ligand_510
	141	ligand_509	111	ligand_510	173	ligand_504	264	ligand_503	68	ligand_509
	196	ligand_506	124	ligand_509	248	ligand_510	288	ligand_509	91	ligand_503
	250	ligand_507	245	ligand_508	295	ligand_507	335	ligand_506	199	ligand_504
	475	ligand_505	343	ligand_505	297	ligand_505	406	ligand_508	211	ligand_505
	488	ligand_501	363	ligand_507	413	ligand_503	480	ligand_510	277	ligand_501
	491	ligand_508	452	ligand_501	416	ligand_501	486	ligand_501	302	ligand_506
	493	ligand_510	509	ligand_504	505	ligand_508	497	ligand_505	358	ligand_508

160		170		180		190		200	
Rank	Ligand	Rank	Ligand	Rank	ligand	Rank	Ligand	Rank	Ligand
10	ligand_506	22	ligand_506	9	ligand_502	98	ligand_509	26	ligand_506
12	ligand_502	37	ligand_503	18	ligand_506	109	ligand_504	60	ligand_504
13	ligand_503	54	ligand_502	40	ligand_504	141	ligand_507	83	ligand_502
27	ligand_509	73	ligand_504	59	ligand_503	153	ligand_502	129	ligand_507
83	ligand_507	215	ligand_507	158	ligand_509	244	ligand_505	169	ligand_509
109	ligand_504	218	ligand_501	278	ligand_510	246	ligand_503	207	ligand_503
140	ligand_510	243	ligand_509	382	ligand_508	259	ligand_506	299	ligand_505
262	ligand_505	391	ligand_508	446	ligand_505	290	ligand_510	432	ligand_510
300	ligand_501	431	ligand_505	453	ligand_501	344	ligand_501	496	ligand_508
449	ligand_508	479	ligand_510	487	ligand_507	465	ligand_508	509	ligand_501

Table S5. Ligand ranking for holo-SrtA NMR structure

MD snapshot (ns)	0		5		10		30		40	
	Rank	Ligand	Rank	Ligand	Rank	Ligand	Rank	Ligand	Rank	ligand
	11	ligand_506	8	ligand_502	6	ligand_502	28	ligand_502	4	ligand_502
	16	ligand_502	71	ligand_506	25	ligand_504	78	ligand_506	21	ligand_505
	47	ligand_504	149	ligand_503	46	ligand_506	144	ligand_509	32	ligand_509
	211	ligand_509	151	ligand_507	68	ligand_509	160	ligand_504	142	ligand_504
	233	ligand_507	182	ligand_509	161	ligand_510	345	ligand_505	170	ligand_507
	240	ligand_505	205	ligand_501	200	ligand_501	369	ligand_510	204	ligand_501
	244	ligand_510	231	ligand_505	241	ligand_503	413	ligand_507	214	ligand_510
	284	ligand_501	240	ligand_504	376	ligand_507	453	ligand_508	274	ligand_503
	337	ligand_508	274	ligand_508	413	ligand_508	479	ligand_503	334	ligand_506
	365	ligand_503	484	ligand_510	489	ligand_505	489	ligand_501	374	ligand_508

	50		60		70		90		100	
	Rank	Ligand	Rank	Ligand	Rank	Ligand	Rank	Ligand	Rank	ligand
	2	ligand_502	4	ligand_502	4	ligand_502	8	ligand_502	4	ligand_506
	55	ligand_508	12	ligand_506	21	ligand_504	17	ligand_506	58	ligand_502
	86	ligand_509	19	ligand_503	76	ligand_505	109	ligand_505	117	ligand_504
	92	ligand_503	130	ligand_505	88	ligand_509	128	ligand_503	128	ligand_508
	95	ligand_501	143	ligand_508	138	ligand_503	165	ligand_508	150	ligand_503
	100	ligand_505	200	ligand_504	172	ligand_508	214	ligand_510	210	ligand_510
	135	ligand_504	216	ligand_509	268	ligand_507	251	ligand_504	220	ligand_509
	164	ligand_506	295	ligand_501	440	ligand_506	293	ligand_507	304	ligand_505
	181	ligand_510	395	ligand_510	467	ligand_510	332	ligand_501	342	ligand_507
	436	ligand_507	409	ligand_507	505	ligand_501	379	ligand_509	411	ligand_501

	110		120		130		140		150	
	Rank	Ligand	Rank	Ligand	Rank	Ligand	Rank	Ligand	Rank	ligand
	7	ligand_502	3	ligand_502	6	ligand_502	1	ligand_506	4	ligand_502
	14	ligand_504	12	ligand_506	8	ligand_506	2	ligand_502	8	ligand_506
	45	ligand_506	53	ligand_504	69	ligand_505	107	ligand_510	80	ligand_504
	60	ligand_509	65	ligand_509	158	ligand_510	123	ligand_509	131	ligand_505
	158	ligand_508	71	ligand_508	185	ligand_508	132	ligand_507	143	ligand_509
	194	ligand_510	84	ligand_510	223	ligand_509	183	ligand_505	173	ligand_503
	239	ligand_507	148	ligand_505	241	ligand_504	288	ligand_504	190	ligand_507
	282	ligand_505	150	ligand_501	249	ligand_501	294	ligand_503	228	ligand_510
	341	ligand_503	162	ligand_507	278	ligand_507	335	ligand_508	281	ligand_508
	456	ligand_501	396	ligand_503	332	ligand_503	391	ligand_501	335	ligand_501

160		170		180		190		200	
Rank	Ligand	Rank	Ligand	Rank	Ligand	Rank	Ligand	Rank	ligand
13	ligand_506	5	ligand_502	1	ligand_502	18	ligand_506	17	ligand_502
15	ligand_502	24	ligand_504	21	ligand_506	29	ligand_502	87	ligand_507
68	ligand_503	59	ligand_509	124	ligand_509	80	ligand_510	98	ligand_505
75	ligand_508	138	ligand_507	138	ligand_505	163	ligand_508	132	ligand_510
98	ligand_509	153	ligand_506	221	ligand_503	207	ligand_507	142	ligand_509
104	ligand_505	184	ligand_508	229	ligand_510	219	ligand_509	159	ligand_504
207	ligand_504	229	ligand_505	232	ligand_508	264	ligand_504	205	ligand_506
218	ligand_507	330	ligand_503	313	ligand_507	308	ligand_505	411	ligand_508
368	ligand_501	409	ligand_510	342	ligand_504	312	ligand_503	469	ligand_503
400	ligand_510	449	ligand_501	414	ligand_501	350	ligand_501	505	ligand_501

Table S6. Performance metrics based on Boltzmann weighted averaged docking scores.

Ensemble	EF1%	BEDROC (α=160.9)	BEDROC (α=20.0)	AUC	RIE
apo-SrtA NMR	0	0.206	0.164	0.52	2.71
holo-SrtA NMR	10	0.283	0.178	0.64	2.94
apo-SrtA crystal	10	0.110	0.151	0.66	2.50
holo-SrtA crystal	0	0.01	0.136	0.61	2.25

Table S7. $EF^{1\%}$, BEDROC (α=160.9), BEDROC (α=20), AUC, RIE values for snapshots of the SMD simulations.

SMD snapshot	$EF^{1\%}$	BEDROC(a=160.9)	BEDROC(a=20.0)	AUC	RIE
1 ns	10	0.284	0.206	0.7	3.41
2ns	10	0.283	0.174	0.69	2.88
3ns	0	0.043	0.144	0.6	2.38
4ns	20	0.392	0.0241	0.68	3.99
5ns	10	0.15	0.122	0.65	2.01
6ns	20	0.494	0.388	0.68	6.41
7ns	10	0.211	0.209	0.76	3.46
8ns	10	0.291	0.199	0.52	3.3
9ns	10	0.286	0.202	0.69	3.34
10ns	10	0.15	0.154	0.64	2.55
11ns	10	0.212	0.263	0.71	4.35
12ns	10	0.151	0.217	0.67	3.58
13ns	10	0.217	0.273	0.71	4.52
14ns	10	0.207	0.283	0.78	4.69
15ns	10	0.151	0.164	0.62	2.71
15.5ns	20	0.464	0.322	0.56	5.33
16ns	10	0.11	0.161	0.65	2.66
16.5ns	20	0.23	0.263	0.68	4.35
17ns	20	0.393	0.308	0.72	5.09
17.5ns	10	0.206	0.139	0.61	2.3
average	12	0.2463	0.2108	0.666	3.737
